# New Insights on the *Zeugodacus cucurbitae* (Coquillett) Bacteriome

**DOI:** 10.3390/microorganisms9030659

**Published:** 2021-03-22

**Authors:** Elias Asimakis, Panagiota Stathopoulou, Apostolis Sapounas, Kanjana Khaeso, Costas Batargias, Mahfuza Khan, George Tsiamis

**Affiliations:** 1 Laboratory of Systems Microbiology and Applied Genomics, Department of Environmental Engineering, University of Patras, 2 Seferi St., 30100 Agrinio, Greece; eliasasim@gmail.com (E.A.); panayotastathopoulou@gmail.com (P.S.); khaesokanjana@gmail.com (K.K.); 2 Laboratory of Applied Genetics and Fish Breeding, Department of Animal Production, Fisheries and Aquaculture, University of Patras, Nea Ktiria, 30200 Messolonghi, Greece; a_sapounas@upatras.gr (A.S.); cbatargias@upatras.gr (C.B.); 3 Institute of Food and Radiation Biology (IFRB), Atomic Energy Research Establishment (AERE), Ganak bari, Savar, Dhaka 1349, Bangladesh; mahfuza79@gmail.com

**Keywords:** melon fly, natural population, symbiome, 16S rRNA gene, next generation sequencing (NGS)

## Abstract

Various factors, including the insect host, diet, and surrounding ecosystem can shape the structure of the bacterial communities of insects. We have employed next generation, high-throughput sequencing of the 16S rRNA to characterize the bacteriome of wild *Zeugodacus* (*Bactrocera*) *cucurbitae* (Coquillett) flies from three regions of Bangladesh. The tested populations developed distinct bacterial communities with differences in bacterial composition, suggesting that geography has an impact on the fly bacteriome. The dominant bacteria belonged to the families Enterobacteriaceae, Dysgomonadaceae and Orbaceae, with the genera *Dysgonomonas*, *Orbus* and *Citrobacter* showing the highest relative abundance across populations. Network analysis indicated variable interactions between operational taxonomic units (OTUs), with cases of mutual exclusion and copresence. Certain bacterial genera with high relative abundance were also characterized by a high degree of interactions. Interestingly, genera with a low relative abundance like *Shimwellia*, *Gilliamella*, and *Chishuiella* were among those that showed abundant interactions, suggesting that they are also important components of the bacterial community. Such knowledge could help us identify ideal wild populations for domestication in the context of the sterile insect technique or similar biotechnological methods. Further characterization of this bacterial diversity with transcriptomic and metabolic approaches, could also reveal their specific role in *Z*. *cucurbitae* physiology.

## 1. Introduction

Symbiosis is the process that occurs when two different organisms live together to form a mutually beneficial partnership. Most insects are symbiotically associated with bacteria [1]. However, the interactions between insects and microorganisms may also be commensal or pathogenic. Microbial symbionts play a significant role in the biology, including nutrition, immunity, reproduction, ecology, and evolution of many insect groups [2,3]. Especially, tephritid flies harbor different bacterial symbionts in their digestive system, which influence different developmental and fitness parameters [4]. This functional contribution of symbiotic microorganisms to insect physiology could find application in mass-rearing facilities, where the manipulation of insects often results in the deterioration of crucial biological parameters. Bacterial strains isolated from stable microbial communities of wild individuals can be provided to mass-reared insects as supplements, in an attempt to replicate the natural microbiome and improve fitness and mating success [5,6,7]. Studies dealing with tephritid microbiomics use either samples from laboratory colonies [8,9,10,11,12,13] or wild populations [10,14,15,16,17,18], which are generally characterized by higher microbial diversity compared to laboratory strains [10,19]. Therefore, it is important to identify and exploit this large portion of diversity, but also to determine how it is affected by the geographical isolation of wild populations.

Fruit flies of the family Tephritidae have a cosmopolitan distribution and are important pests in fruit production [20]. The genus *Bactrocera* is highly diverse and abundant, with many species still to be described [21]. At least 28 *Bactrocera* subgenera have been described and these are divided into four groups, namely *Bactrocera*, *Melanodacus*, *Queenslandacus*, and *Zeugodacus* [22].

*Zeugodacus* currently includes 192 species [23]. At least 50% of the species included in the *Zeugodacus* group, for which host plant records are available, are cucurbit feeders [23]. Amongst them, the melon fly, *Zeugodacus* (*Bactrocera*) *cucurbitae* (Coquillett), is of particular economic importance. Recently the systematic position of *Zeugodacus* was revised as *Bactrocera*, *Dacus* and the subgenera of the *Zeugodacus* group have different evolutionary histories [24,25]. In this study, we refer to the classification proposed by Virgilio et al. (2015) by using the new generic combination *Zeugodacus* (*Zeugodacus*) *cucurbitae* for the melon fly, although most existing literature refers to it under the former combination, *Bactrocera* (*Zeugodacus*) *cucurbitae*.

Even though *Z. cucurbitae* was initially described from the Hawaiian Islands, its presence was the result of accidental human-mediated introduction [26]. Nowadays, it is distributed across a range of climatic regions such as Central and East Asia (including Pakistan, India, Bangladesh, Nepal, China, Indonesia and the Philippines) and Oceania (including New Guinea and the Mariana Islands) [27]. Due to its vast adaptability, high reproduction potential and invasion capacity, the melon fly has been the subject of a worldwide pest management program [28].

Culture-dependent and culture-independent approaches have been employed to characterize the structure of the symbiotic community of Tephritidae species, including the melon fly [5,29,30,31]. Hadapad et al. (2015) investigated the composition and diversity of the microbial community in the midgut of melon fly, obtained from nine wild populations (India), and resulted in the dominant species inhabiting the midgut of the melon fly, which were from the genera *Enterobacter* (34.6%), *Klebsiella* (19.2%), *Citrobacter* (7.7%), *Bacillus* (15.4%) and *Providencia* (7.7%), and 3.8% each of *Micrococcus*, *Staphylococcus*, *Leclercia* and *Exiguobacterium* [30]. Another reported study on cultivable microbiota of *Zeugodacus cucurbitae* was performed by Gujjar et al. and shed light on a distinct pattern of gender specific gut bacterial colonization. The cultivable diversity from females of *Z. cucurbitae* comprised mainly of *Morganella morganii* and *Bacillus pumilis* while *Z. cucurbitae* males were predominantly colonized by aerobic endospore formers viz., *Bacillus cereus*, *B*. *licheniformis* and *B. subtilis* [32].

Beyond the insect host and its diet, the insect microbiota may be interconnected with the surrounding ecosystem. The present study aimed to investigate the structure of the bacterial symbiome of *Z. cucurbitae* flies in different locations distributed in Bangladesh, trying to reveal any association between the insect’s bacterial community profile and their environment. The Illumina MiSeq platform for high-throughput sequencing of 16S rRNA gene was utilized to analyze and characterize the whole fly bacteriome.

## 2. Materials and Methods

### 2.1. Z. Cucurbitae Collection and Storage

Male adult *Z. cucurbitae* flies were collected from three locations (Table 1) in Bangladesh during the period of May and June 2013, with the use of cue-lure traps, which contained highly attractive kairomone lures for male melon flies [33]. The collected melon flies were placed individually in plastic vials to prevent cross-contamination of bacteria between flies of the same location as well as from different locations. Collected flies were preserved in vials containing 800 μL of absolute ethanol to protect them from regurgitation within the tubes, and stored at −20 °C.

### 2.2. DNA Extraction, 1st Step PCR Amplification and Purification

The selected samples were firstly surface sterilized with sterile water and thereupon transferred to new tubes containing lysis buffer for grinding with a homogenizer. Total DNA extraction was performed following a simplified CTAB protocol [34]. The quality of the DNA preparations and the concentration of double-stranded DNA were both analyzed by microvolume spectrophotometry.

Polymerase chain reaction (PCR) was performed with KAPA HiFi HotStart ReadyMix PCR Kit (KAPA BioSystems, USA) and the previously extracted DNA as a template. The variable V3–V4 region of the bacterial 16S rRNA gene sequences was amplified with the primer pair U341F-MiSeq 5′-CCT ACG GGR SGC AGC AG-3′ and 805R-MiSeq 5′-GA CTA CHV GGG TAT CTA ATC C-3′ [35]. Each 25 μL reaction contained 5μL of KAPA HiFi Fidelity Buffer (5X), 0.7 μL of dNTPs solution (10 mM each), 0.7 μL of each primer solution (10 μM), 0.3 μL of KAPA HiFi HotStart DNA Polymerase solution (1 U/μL), 1 μL from the template DNA solution and was finalized with 16.6 μL of sterile deionized water. The PCR amplifications were performed with a 3-min incubation at 95 °C followed by 30 cycles of 98 °C for 20 s, 60 °C for 15 s and 72 °C for 45 s, and a final 1-min extension at 72 °C. Negative and positive controls were always performed in parallel. All PCR products were separated in a 1.5 % (*w/v*) agarose gel in TAE buffer (40 mM Tris–acetate, 1 mM EDTA). The desired approximately 550 bp amplification product was visualized in Bio-Rad’s Gel Doc XR+ system. Positive PCR products were purified from unincorporated primers and nucleotides with a 20 % PEG, 2.5 M NaCl solution, centrifuged at 14,000× *g* for 20 min and the precipitate was washed twice with 125 μL of a 70 % *v/v* ethanol solution and centrifuged at 14,000× *g* for 10 min. The dried precipitates were suspended in 15 μL of sterile deionized water and the concentration was measured with a Quawell Q5000 micro-volume UV−Vis spectrophotometer.

### 2.3. Index PCR Amplification and Purification

The resulting PCR amplicons were diluted up to 10 ng/μL and then used as templates within the second-step PCR for further amplification, and to include the indexes (barcodes) as well as the Illumina adaptors. The combinatorial use of index primers resulted in unique samples that were pooled and sequenced on one Illumina MiSeq run. In more detail, amplification reaction was performed using the KAPA HiFi HotStart PCR Kit in a final volume of 50 μL. Each reaction contained 10 μL of KAPA HiFi Fidelity Buffer (5×), 1.5 μL of dNTPs solution (10 mM each), 5 μL of the forward indexing primer (10 μM), 5 μL of the reverse indexing primer (10 μΜ), 1 μL of KAPA HiFi HotStart DNA Polymerase (1 U/μL), 2 μL from the diluted PCR product (10 ng/μL) and 25.5 μL of sterile deionized water. The PCR amplifications were performed with a 3-min incubation at 95 °C followed by 8 cycles of 95 °C for 30 s, 55 °C for 30 s and 72 °C for 30 s, and a final 5-min terminator reaction at 72 °C. The resulting amplicons from indexing PCR were purified using Macherey-Nagel’s NucleoMag^®^ NGS Clean-up and Size Selection kit according to the manufacturer’s recommendations. Amplicons from different samples were quantified with a Quawell Q5000 micro-volume UV−Vis spectrophotometer and merged in equimolar ratios (8 nM).

### 2.4. NGS Sequencing and Bioinformatics Analysis

Sequencing was performed on the Illumina MiSeq platform, in 300 bp paired-end runs, by Macrogen Inc. (Seoul, Korea). Raw sequencing reads were demultiplexed, converted to FASTQ, and the Illumina adapters were trimmed using Illumina standard algorithms. Bioinformatic analysis of raw sequencing reads was conducted in usearch v. 11 [36]. Paired-end reads were assembled, trimmed by length and further corrected for error and quality using the -fastq_mergepairs option. The quality of the assembled sequences was further improved using -fastq_filter, followed by identifying unique read sequences and abundances with the -fastx_uniques option. Sequences were clustered into Operational Taxonomic Units (OTUs) using the -cluster_otus command [37]. Clustering was performed at 97, 94, 90 and 81% sequence similarity. Cross-talk errors were identified and filtered with –uncross option based on the UNCROSS2 algorithm [38]. Taxonomy was assigned with Qiime2 [39] using the BLAST+ algorithm against the SILVA 128 release database [40].

Alpha diversity analyses were performed with the “diversity” function of the R package “vegan”, and alpha diversity indices were plotted using “ggplot” function from package “ggplot2”. Statistical differences in bacterial composition and relative abundance between populations were detected using the non-parametric Kruskal−Wallis Rank Sum Test along with Wilcoxon Rank Sum Test [41]. Beta-diversity analysis was calculated using generalized UniFrac [42]. Visualization of the multidimensional distance matrix in a space of two dimensions was performed by the robust nonmetric version of Multi-Dimensional Scaling (NMDS) [43]. A permutational multivariate analysis of variance using distance matrices was performed with the function “adonis” of the package “vegan” in order to determine if the separation of groups was significant, as a whole and in pairs [44]. The 16S rRNA sequences reported in this study have been deposited in NCBI under Bioproject number PRJNA701541.

Interactions between microorganisms were investigated and visualized through co-occurrence networks. These interactions refer to microorganisms performing similar or complementary functions and/or sharing similar environmental conditions, but not necessarily having physical interactions [45,46] Network analysis of OTUs was performed using the CoNet plugin [47] in Cytoscape 3.8.2 (Institute for system biology, Seattle, WA, USA), and co-occurrence graphs were obtained using Gephi 0.9.2 (Gephi, WebAtlas, Paris, France). To build the network, the Pearson and Spearman correlation coefficients, Mutual Information, and the Bray–Curtis and Kullback–Leibler dissimilarity indices were combined. To compute the statistical significance of the copresence/mutual exclusion, edge-specific permutation and bootstrap score distributions were calculated with 1000 iterations. Edges with original scores outside the 0.95 range of their bootstrap distribution were discarded, and p-values were corrected using the Benjamini–Hochberg method. Nodes in each network visualization corresponded to microbial OTUs and edges to the microbial associations. The size of each node was proportional to the degree of interactions.

## 3. Results

### 3.1. 16S rDNA Sequence Reads

In the present study, the microbiota diversity across three wild *Z. cucurbitae* populations from Bangladesh, was unraveled utilizing a deep-sequencing approach on the Illumina MiSeq platform. In total 60 samples were analyzed, 20 from each population. After demultiplexing, quality filtering and chimera removal, the data set consisted of 1.27 million high-quality paired end sequences. Samples contained on average 21,273 sequences that were divided into 523 OTUs. In total, 47 OTUs contained more than 0.1% of total sequences and were kept for the rest of the analysis (Supplementary Table S1). Based on 97% sequence similarity, 28 OTUs were classified into three phyla, four classes and 15 genera, while 19 remained unassigned and were classified at lower similarities (Supplementary Table S2). Seven remained unclassified at 94 and two at 90% sequence similarity. These unassigned OTUs could potentially belong to new uncharacterized taxa. Based on 81% sequence similarity, all OTUs classified into three phyla, four classes and 20 genera.

### 3.2. The Environment Shapes the Insect Bacteriome

#### 3.2.1. Bacterial Diversity between Wild Populations

**Figure 1:**
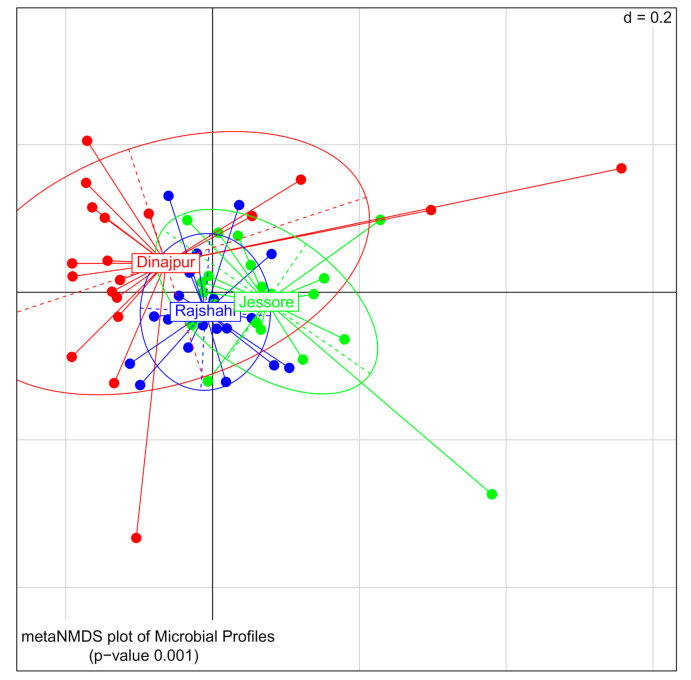
Nonmetric multidimensional scaling (NMDS) plot of bacterial communities for *Z*. *cucurbitae* samples collected from Dinajpur (red), Jessore (green) and Rajshahi (blue) (*p <* 0.001). The ‘d’ indicates dissimilarity scale of the grid (d = 0.2 mean that the distance between two grid lines represents approximately 20% dissimilarity between the samples).

Beta-diversity analysis revealed that bacterial communities of wild *Z. cucurbitae* were clustered according to the geographic origin of their population. The NMDS plot based on generalized UniFrac showed that even though groups overlapped, they differed significantly (PERMANOVA, *p <* 0.001, Figure 1). The same picture was also observed after pairwise comparison between the three territories (PERMANOVA, *p <* 0.002, Supplementary Figure S1).

#### 3.2.2. Comparing Alpha-Diversity between Different Populations

**Figure 2:**
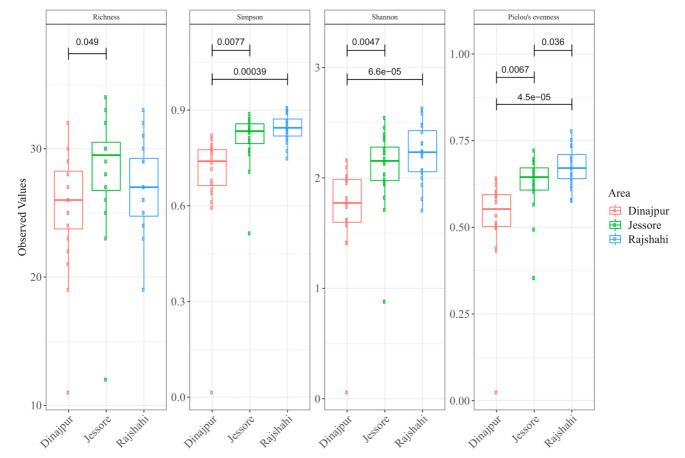
Species richness and diversity indices with significance differences in natural populations of *Z*. *cucurbitae* samples collected from the regions of Dinajpur, Jessore, and Rajshahi. Boxes represent interquartile range (IQR), the line within the boxes is the median, and the dots represent samples.

Differences in bacterial richness and diversity were observed between the three wild populations (Figure 2). The population from Jessore exhibited the highest richness and the population from Rajshahi the highest diversity. In terms of richness, Jessore showed marginally significantly higher values than Dinajpur (pairwise Wilcoxon Rank Sum Test: adjusted *p <* 0.05) but not Rajshahi. Additionally, based on Simpson and Shannon indices, bacterial diversity was significantly higher in Jessore and Rajshahi compared to Dinajpur (pairwise Wilcoxon Rank Sum Test: adjusted *p <* 0.05). Pielou’s eveness index also showed a statistical difference even between Rajshahi and Jessore (pairwise Wilcoxon Rank Sum Test: adjusted *p <* 0.05).

#### 3.2.3. Bacterial Composition of Wild *Z. cucurbitae* Populations

**Figure 3:**
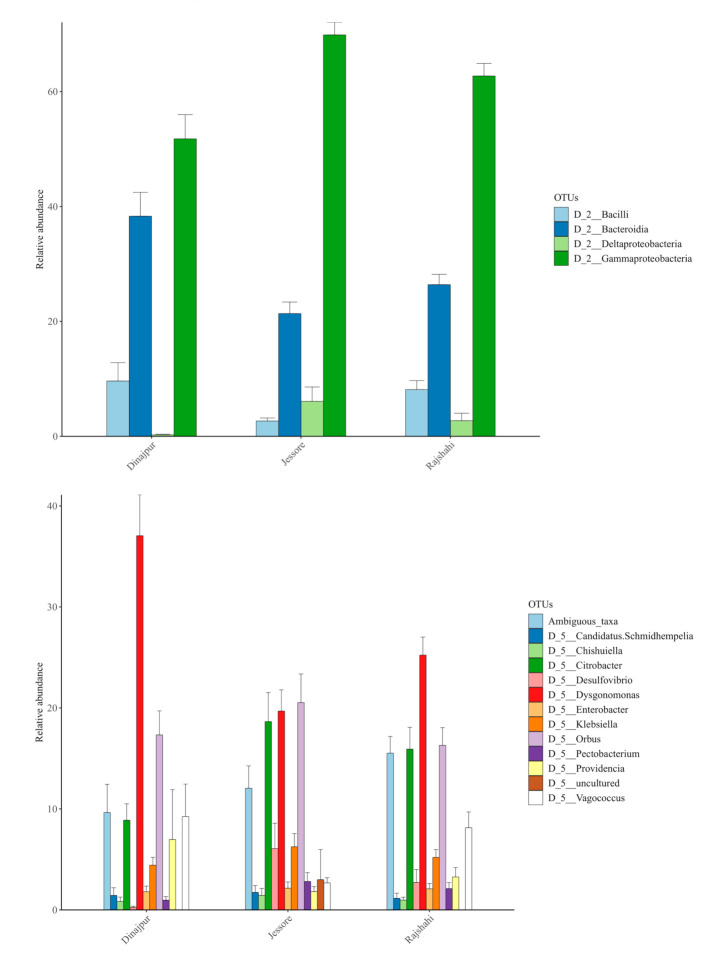
Relative abundance of wild *Z. cucurbitae* microbiota at the Class (D_2) and Genus (D_5) Level.

**Figure 4:**
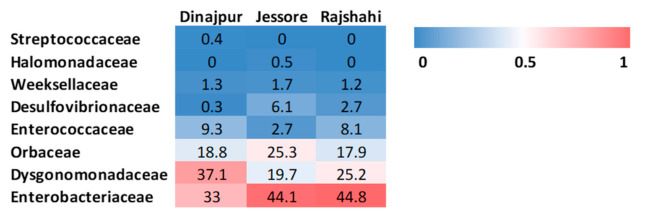
Heat map of bacterial families identified in populations of *Z. cucurbitae* from Bangladesh.

**Figure 5:**
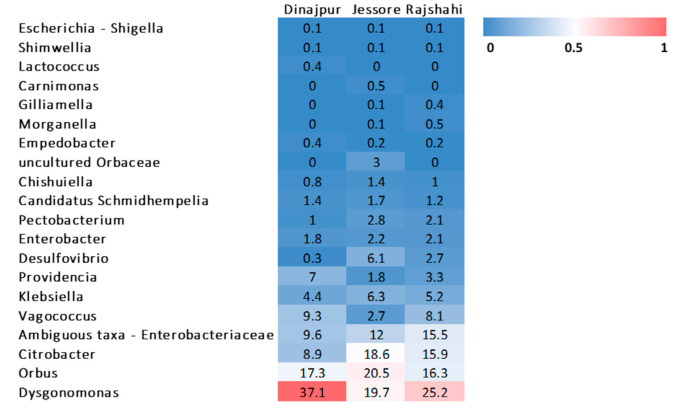
Heat map of bacterial genera observed in wild populations of *Z. cucurbitae* from Bangladesh.

Sequences from three bacterial phyla were present in all wild *Z. cucurbitae* populations. All three territories, Dinajpur, Jessore and Rajshahi, showed similar composition at phylum level dominated by Proteobacteria (52.0 ± 4.2, 76.0 ± 2.1 and 65.5 ± 2.3%, respectively), Bacteroidetes (38.3 ± 4.1, 21.4 ± 2.0 and 26.4 ± 1.8%, respectively) and Firmicutes (9.6 ± 3.2, 2.7 ± 0.5 and 8.1 ± 1.6%, respectively). Firmicutes and Bacteroidetes were each represented by a single class, Bacilli and Bacteroidia, respectively (Figure 3). Two classes were found in Proteobacteria. The most abundant were Gammaproteobacteria (51.8 ± 4.2 Dinajpur; 69.9 ± 2.2 Jessore and 62.7 ± 2.2% Rajshahi), followed by a smaller portion of Deltaproteobacteria (0.3 ± 0.1 in Dinajpur; 6.1 ± 2.5 in Jessore and 2.7 ± 1.3% in Rajshahi) (Figure 3). Sequences that belonged to the families, Desulfovibrionaceae, Dysgonomonadaceae, Enterobacteriaceae, Enterococcaceae, Halomonadaceae, Orbaceae, Streptococcaceae and Weeksellaceae were identified (Figure 4). Among them, the most prevalent were Enterobacteriaceae (40.6 ± 3.9), Dysgonomonadaceae (27.3 ± 2.6), Orbaceae (20.6 ± 2.5) and Enterococcaceae (6.7 ± 1.7). At the genus level, *Dysgonomonas* was the most frequent in Dinajpur and Rajshahi (37.1 ± 4.0 and 25.2 ± 1.8%), and *Orbus* in Jessore (20.5 ± 2.8%), although *Dysgonomonas* retained high relative abundance in those samples too (19.7 ± 2.1%) (Figure 3 and Figure 5). The majority of OTUs were present in all three populations with varying frequencies, except for three genera (Supplementary Table S1), among them, an uncultured genus from the family Orbaceae (OTU13) that was only identified in Jessore (3.0 ± 3.0%). Ambiguous taxa, on the other hand, which are present in all populations (average 12.4 ± 2.2%), contain sequences that belong to an unclassified genus of the Enterobacteriaceae family (OTU4). Pairwise comparisons between populations revealed significant differences in the relative abundance of 14 OTUs (pairwise Wilcoxon Rank Sum Test; adjusted *p*-value < 0.05) (Supplementary Figure S2). Among them, sequences identified as *Enterobacter* (OTU123) were significantly more abundant in the population from Dinajpur compared to the other two and *Klebsiella* were significantly higher in Dinajpur and Jessore compared to Rajshahi (OTU45) (pairwise Wilcoxon Rank Sum Test; adjusted *p*-value < 0.05).

#### 3.2.4. Mutual Exclusion/Co-Occurrence Network Analysis

**Figure 6:**
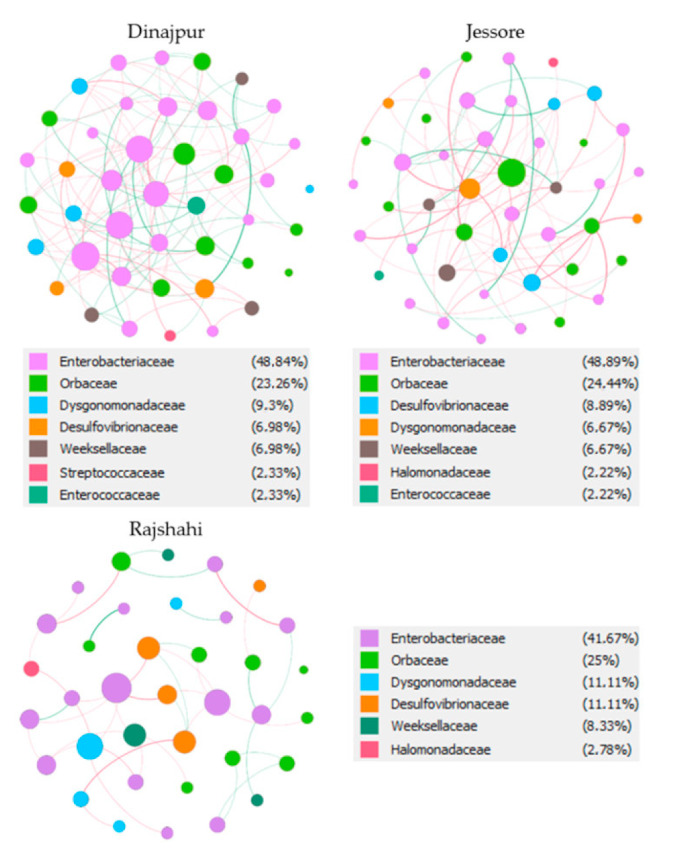
Mutual exclusion and co-occurrence networks at the family level for the OTUs that compose the bacterial communities of wild adult *Z. cucurbitae* flies from Bangladesh. The size of each node is proportional to the degree of interactions. Green edges represent cases of copresence and red edges mutual exclusion. The numbers in parentheses next to each OTU describe the percentage of nodes that belong to each family in the network.

Potential interactions between bacterial partners of each region were investigated with co-occurrence and mutual exclusion network analysis. The networks for each region were visualized at the family (Figure 6) and genus level, and detailed information about their topology is included in Supplementary Figure S3, Supplementary Tables S3 and S4. The three areas were characterized by a different number of OTUs (nodes), interactions (edges) and clustering coefficients. Dinajpur and Jessore were characterized by almost the same number of nodes (43 and 45, respectively), which was higher than Rajshahi (36 nodes). Moreover, bacterial communities from Dinajpur showed more interactions (113) and lower clustering coefficient (0.205) compared to Jessore (77 edges and 0.349 coefficient) and Rajshahi (40 edges and 0.291 coefficient). Most of the interactions were classified as mutual exclusions (59–78%), while 22–41% were cases of copresence. Proteobacteria dominated in terms of interactions in all three areas. At the genus level, Pectobacterium (OTU16 and OTU20) showed the highest degree of interactions in Dinajpur, Orbaceae (OTU13) and Dysgonomonas (OTU9) in Jessore, and Klebsiella (OTU281) with Enterobacter (OTU123) in Rajshahi.

## 4. Discussion

The aim of this study was to investigate the bacterial community structure of wild *Z. cucurbitae* flies that were collected in three districts of Bangladesh and assess the geographical impact on its composition. The analysis of the community was based on high throughput sequencing of the V3–V4 section of the bacterial 16S rDNA.

Taxonomic assignment of sequences revealed a large portion of uncharacterized bacterial diversity that resides within these flies. After OTU classification at 97%, 19 OTUs remained unassigned. Twelve of them were classified at 94% sequence similarity, five at 90% and two at 81%. Although the sequence similarity check is restricted at 460 bp and not the whole 16S rDNA, this result may still suggest the presence of new bacterial species, genera, and families. It is noteworthy that 40% (19 out of 47) of bacterial OTUs identified in the wild *Z. cucurbitae* could potentially belong to new families (2 out of 19), genera (5 out of 19) or species (12 out of 19), suggesting that there is a large portion of unexplored diversity in these populations. Characterizing this hidden bacterial diversity could help us identify important components of the melon fly bacteriome or taxa with interesting overall properties. This knowledge could prove useful, especially when we want to reconstruct the bacterial community of wild populations that have been introduced to the lab for mass-rearing purposes, in order to improve fitness and mating success. The process of colony adaptation typically results in the development of distinct bacterial communities between wild and laboratory populations, with laboratory populations usually experiencing a loss in diversity [10,19,48,49]. This could lead to the loss of important bacteria for the physiology of the mass-reared flies and a subsequent loss of fitness. Sex related differences in the microbiota have been documented in both wild and laboratory populations of the fly [10,13]. In this work however, only male subjects were analyzed, due to the fact that samples were collected using traps with male attractants.

Results suggest that the composition of the bacterial communities was significantly associated to the origin of the population. Changes in the structure of microbial communities among populations with different geographic distributions have been recorded in other *Z. cucurbitae* populations [16,50], but also in other fly species or insects [18,19,51,52,53]. Seasonal changes in climate conditions may shift the composition of the insect microbiome [54]. In our study, sampling took place within a short period (May−June) with fairly stable environmental conditions in all three areas. In order to better assess the effect of climate on the microbial profile, sampling could be performed within a longer period with seasonal weather variations. Other parameters affecting this differentiation could be related to host availability/adaptation or diet, since diet plays an important role in shaping the intestinal bacterial community of flies [49,55,56,57]. The diet of adult flies mainly includes plant exudates, honeydew and bird droppings scattered on leaves and fruits [58], while larvae develop in cucurbits [23]. This difference in dietary habits is reflected in the distinct microbial communities between developmental stages of the fly, especially between larval and adult stages [50,59]. However, regarding larvae, their microbiome did not seem significantly affected by different host plants [50]. In our case, since samples were collected with traps placed in orchards with various cucurbit crops, it was difficult to assess the specific adult diet or larval host, and therefore their impact on the identified bacterial communities.

More specifically, flies from Rajshahi and Jessore exhibited higher diversity and richness than Dinajpur. Proteobacteria, were the prevalent phylum in all samples, followed by a significant portion of Bacteroidetes. So far, Proteobacteria seem to be the prevalent phylum in *Z. cucurbitae* flies, whether they originate from laboratory or wild populations [10,13,16]. However, their relative abundance varies. Hadapad et al. identified higher relative abundance (87.7%) in wild *Z. cucurbitae*, from Karnataka, India, compared to the average relative abundance of our populations (64.5%) and even to the population with the highest percentage (76% in Jessore). Yong et al. [16] found on average 75.8% (59.82 to 97.69%) in three populations from Thailand, Peninsular Malaysia, and Sarawak.

Such differences could be explained by the impact of geography, host availability (different cucurbit hosts, e.g., pumpkin, bitter gourd, sweet gourd, bottle gourd, cucumber, etc.) or dietary habits. Other factors that might contribute to these differences are technical issues, such as the nature of samples (whole flies or specific tissue used, e.g., gut tissue), sample preservation and handling, or the number of reads per sample acquired during sequencing. For instance, it has been observed that preservation in ethanol has an impact on the resulting microbiome profiles, affecting the presence of both dominant and less represented OTUs [60].

Proteobacteria are generally associated with the gastrointestinal tract of flies, so their relative abundance is expected to increase when only the gut tissue is considered. This phylum was also dominant in mass-reared *Z. cucurbitae* from India (64.15%) [10] and Bangladesh (97%) [13]. Similarly, Bacteroidetes was the second most frequent phylum in all studies (21.4–38.3 our study; ~3–17 Hadapad et al.; 8.55–22.76 Yong et al.) and Firmicutes the third (2.7–9.6 our study; ~1.5–2.5 Hadapad et al.; 3.44–14.05 Yong et al.). Hadapad et al. also identified the presence of Cyanobacteria/Chloroplast but with very low relative abundance (<0.5%), while Yong et al. found traces of Actinobacteria (<0.01%). It seems that *Z. cucurbitae* flies contain a microbiome that consists of bacterial strains belonging to three phyla, Proteobacteria, Bacteroidetes and Firmicutes, with this specific order in terms of prevalence, but varying relative abundances. These phyla are also very common components of the microbiome of closely related *Bactrocera* species [11,14,18,61,62,63].

At family level, *Z. cucurbitae* populations exhibit differences. Our samples were characterized by the presence of Enterobacteriaceae, Dysgonomonadaceae, Orbaceae and Enterococcaceae. Yong et al. found 39 families across all samples, with six of them, namely Porphyromonadaceae, Enterococcaceae (3.5–14.1) Burkholderiaceae, Enterobacteriaceae, Pectobacteriaceae, and Orbaceae (7.4–14.7), being present in all three areas. Enterobacteriaceae was the prevalent family (43–67%). Dysgonomonadaceae was only found in very low frequencies, 0.2–1.4% [16]. The population from India mostly contained Enterobacteriaceae (dominant, 68.7%), Flavobacteriaceae, Brucellaceae, Pseudomonadaceae, Sphingobacteriaceae and Streptococcaceae. Enterococcaceae and Orbaceae were present but with relatively low frequencies, while Dysgonomonadaceae were not detected [10]. Enterobacteriaceae is usually among the dominant families in closely related *Bactrocera* or other fly species [11,14,18,19,31,49,61,62,63,64,65,66,67].

Bacteria of the genus *Dysgonomonas* showed the highest overall prevalence in the studied populations from Bangladesh. They were dominant in two populations and second only to *Orbus* in Jessore. Yong et al. identified the genus in all studied populations from Thailand, and Malaysia [16], but with low relative abundance (0–3.2%). In contrast, they were not found in wild *Z. cucurbitae* from India, but were highly abundant in mass reared adult flies [10]. A low presence of *Dysgonomonas* was also detected in wild populations of the relative species *Bactrocera dorsalis* from Hainan and Guizhou provinces in China [18]. A very low presence was detected in different developmental stages of *B. dorsalis* from Huizhou and Nansha from China [31]. *Dysgonomonas* sequences were also identified in all developmental stages of *B. dorsalis* from Wuhan [15]. Predominance of *Dysgonomonas* bacteria has been found in certain insects, such as wild *Phormia regina* flies (36.8%) [68] and bombardier beetles, *Brachinus elongatulus* (5.6–54.4%) [69]. *Dysgonomonas* are facultatively anaerobic cocci that produce acids by fermenting several carbohydrates [70]. Representatives of the genus that were isolated from the digestive tract of fungus-growing and wood-feeding species of termites exhibited such hydrolytic activity [71,72]. Genome sequencing and preliminary analysis of one of the isolated strains, revealed the presence of an array of hydrolytic enzymes related to decomposing lignocellulose, and carbohydrate degradation in general [73]. It is therefore possible that the bacteria are related to nutrition also in *Z. cucurbitae* flies, by participating in sugar fermentation.

The genus *Orbus* was overall the second most frequent in the studied populations. It was also among the dominant genera in wild populations from Thailand, and Malaysia, occurring in all populations with relative abundance values between 0–24.2% [16]. On the other hand, the bacteria were absent from Indian populations either when tested with high throughput sequencing [10] or culture dependent approaches [30,74]. *Orbus* bacteria were found in the closely related species from Chinese populations with 13.8–42.8% relative abundance [18], in *B. dorsalis* from Wuhan (0.04%) [15], but also in *B. tryoni* (Froggatt) (2%) and *B. cacuminata* (4.8%) from Australia [61]. The genus was also found in wild-collected Drosophila (29%) [49] and butterflies (9.8%) [75,76]. A Gram-negative, facultatively anaerobic, coccoid *Orbus* strain was isolated from the gut of the butterfly *Sasakia charonda* and exhibited similar carbohydrate hydrolytic activity to the *Dysgonomonas* bacteria mentioned previously [77]. The closely related Orbaceae species *Gilliamella apicola*, also provide their insect hosts, honeybees (Apis) and bumble bees (Bombus), with fatty acids by degrading plant carbohydrates [78]. An additional role to their host physiology is the detoxification of poisonous sugars [79].

As expected, many members of the dominant Enterobacteriaceae family were identified in our *Z. cucurbitae* samples. Among them *Citrobacter* were the most frequent, followed by *Klebsiella*, *Providencia*, *Enterobacter* and *Pectobacterium*. All of them were also identified in Thai and Malay populations with varying relative abundance [16]. In this case however, *Klebsiella* showed higher values (14–61.3%) compared to *Citrobacter* (0.4–35.2%), *Providencia* (0–9.4%), *Pectobacterium* (0.02–5.6%), and *Enterobacter* (0.07–0.5%). It is notable that only *Providencia* were identified in the samples from India [10], with this result probably reflecting differences in geographical origin, host availability and adaptation. However, four of them, were found in populations from India by culture dependent approaches, specifically *Enterobacter* (34.6%), *Klebsiella* (19.2%), *Citrobacter* (7.7%) and *Providencia* (7.7%) [30]. Isolates belonging to *Enterobacter*, *Klebsiella*, and *Citrobacter* were also found in another Indian population [74]. *Klebsiella*, *Citrobacter*, *Pectobacterium* and *Enterobacter* bacteria are mostly related to nitrogen fixation or exhibit pectinolytic activity. In this way, they provide their hosts with the nitrogen resources required to synthesize essential amino acids that they are otherwise unable to receive from their nitrogen-poor diet [64,65,80]. Some strains have also probiotic properties when provided as food supplements in mass-rearing facilities and could be used for improving important fitness parameters of hosts [5,6,8,81,82,83,84,85]. On the other hand, certain *Providencia* bacteria exhibit pathogenicity in insects [86,87]. However, their complete role in insects has not been fully deciphered, and thus a positive impact of these bacteria cannot be excluded.

Sequences belonging to *Vagococcus* and *Desulfovibrio* were also identified in our study. *Vagococcus* were overall more frequent, except for the population from Jessore. Yong et al. also identified both genera in all studied *Z. cucurbitae* populations, however, with different mean relative abundances (0–0.03% for *Vagococcus* and 0–28.1% for *Desulfovibrio*) [16]. *Vagococcus* were also detected in wild samples from India, while *Desulfovibrio* were absent [10]. Even though they are uncommon, these bacteria are present in other related species. *Vagococcus* sequences were present in *B. dorsalis* and *B. minax* from regions of China, with various frequencies, ranging from 0.001–11% [11,15,18,19,31,66]. On the other hand, *Desulfovibrio* are less common in *B. dorsalis*, with very low relative abundance (less than 0.1%) [31]. Similar to members of the Enterobacteriaceae, *Desulfovibrio* bacteria are possibly related to nutrition through nitrogen fixation for their hosts [64,80]. Bacteria of the genus *Vagococcus* have been isolated among others, from wasps [88], mosquitoes [89] and various species of flies [90,91]. Even though their importance in insect host physiology has not been fully explored, they have been shown to inhibit La Crosse virus in *Aedes albopictus* mosquitoes in vitro [92]. Some strains exhibit pathogenicity in the rainbow trout, *Oncorhynchus mykiss* [93], while others exhibit a probiotic effect, by stimulating the immune system of aquaculture fish or by protecting them from known pathogens, e.g., *Vibrio anguillarum*, a similar effect to that described in mosquitoes [94,95].

Network analysis indicated that the community from Dinajpur developed more interactions compared to the other two regions. Interestingly, certain OTUs with low relative abundance (even below 1%), like *Shimwellia* (OTU52), *Gilliamella* (OTU17), *Chishuiella* (OTU12), uncultured Orbaceae (OTU13) and *Candidatus* Schmidhempelia (OTU10) developed a high number of interactions, suggesting that even low represented OTUs may have an important role in the symbiotic community. For example, in the case of Dinajpur, even though *Shimwellia* (OTU52) and *Escherichia*-*Shigella* (OTU319) show only 0.1% relative abundance, they exhibit the highest number of positive interactions in the community.

## 5. Conclusions

Geographic distribution was clearly associated with the composition of bacterial communities in wild *Z. cucurbitae* flies from Bangladesh. All three studied districts/territories had developed distinct bacterial communities. However, the samples contained specific bacterial genera. The predominant were *Dysgonomonas*, *Orbus*, *Citrobacter*, *Vagococcus*, *Klebsiella*, *Providencia*, *Desulfovibrio* and *Enterobacter*. All genera were identified in previously studied populations from Thailand and Malaysia, but with differences in relative abundance. Most of these bacteria are related to nutrition in various insect species, except for Providencia that are generally pathogenic. Further characterization of this bacterial diversity with transcriptomic or metabolomic analyses, could shed light on their specific role in *Z. cucurbitae* natural populations. This metabolic potential, if it exists in *Z. cucurbitae* flies, could be exploited with the aim of improving its fitness during mass-rearing for SIT purposes.

## Figures and Tables

**Table 1:** Sampling locations in Bangladesh and the number of samples that were collected from each location.

Region	Location	Coordinates		Number of Insects
		Latitude	Longitude	Male
Dinajpur	Northwest	25.819010	88.649265	20
Jessore	Southwest	23.177112	89.180159	20
Rajshahi	Northwest	24.489453	88.612312	20

## Data Availability

Data sharing is not applicable to this article.

## Supplementary Materials

The following are available online at https://www.mdpi.com/2076-2607/9/3/659/s1. Figure S1: Pairwise non-metric multidimensional scaling (NMDS) plot of bacterial communities for *Z*. *cucurbitae* samples collected from Dinajpur (red), Jessore (green) and Rajshahi (blue). All p-values are below 0.05 indicating statistically significant differences. ‘d’ indicates dissimilarity scale of the grid (d = 0.2 mean that the distance between two grid lines represents approximately 20% dissimilarity between the samples); Figure S2: Alpha diversity indices and pairwise comparisons of OTUs that show statistically significant differences (*p*-value < 0.05) among the three tested areas; Figure S3: Mutual exclusion and co-occurrence networks among OTUs (genera) in the three tested areas; Table S1: Number of reads and the relative abundance of each OTU in the three tested populations; Table S2: Nineteen OTUs remained unassigned and were classified at lower similarities. These unassigned OTUs could potentially belong to new uncharacterized taxa. The column titled “Taxonomy assigned at” contains the % similarity threshold that each sequence was classified during taxonomy assignment; Table S3: Network statistics for each region; Table S4: Network statistics for the OTUs in the three tested areas. The column titled “degree” contains the total number of interactions. The column titled “posdegree” contains the number of positive interactions, and “negdegree” negative interactions.
