# Supplementary material for: New Insights on the Zeugodacus cucurbitae (Coquillett) Bacteriome
Source: Microorganisms. 2021 Mar 22;9(3):659. doi: 10.3390/microorganisms9030659 (PMC8004655; doi:10.3390/microorganisms9030659)
Supplement: Supplementary file 1 [file microorganisms-09-00659-s001.pdf]

Supplementary File

Supplementary Table S1: Number of reads and the relative abundance of each OTU in the three tested populations

		Number of reads			Relative abundance		
**#OTU**	**Genus**	**Dinajpur**	**Rajshahi**	**Jessore**	**Dinajpur**	**Rajshahi**	**Jessore**
Otu1	*Dysgonomonas*	350132	196943	180832	0.3667	0.2046	0.1896
Otu10	*Candidatus* Schmidhempelia	11283	10284	14942	0.0118	0.0107	0.0157
Otu104	*Klebsiella*	2467	1691	1371	0.0026	0.0018	0.0014
Otu11	*Pectobacterium*	2658	9798	17903	0.0028	0.0102	0.0188
Otu119	*Orbus*	760	2252	1457	0.0008	0.0023	0.0015
Otu12	*Chishuiella*	7872	9357	13924	0.0082	0.0097	0.0146
Otu123	*Enterobacter*	3599	680	503	0.0038	0.0007	0.0005
Otu13	uncultured Orbaceae	0	0	26511	0.0000	0.0000	0.0278
Otu14	*Orbus*	7578	17776	12366	0.0079	0.0185	0.0130
Otu15	*Dysgonomonas*	960	13710	1169	0.0010	0.0142	0.0012
Otu16	*Pectobacterium*	1898	8014	5608	0.0020	0.0083	0.0059
Otu17	*Gilliamella*	227	4232	914	0.0002	0.0044	0.0010
Otu18	*Carnimonas*	0	346	4351	0.0000	0.0004	0.0046
Otu19	*Morganella*	238	4873	502	0.0002	0.0051	0.0005
Otu2	*Orbus*	86950	100386	151112	0.0911	0.1043	0.1584
Otu20	*Pectobacterium*	4610	2458	3712	0.0048	0.0026	0.0039
Otu200	*Candidatus* Schmidhempelia	2147	719	1617	0.0022	0.0007	0.0017
Otu21	*Desulfovibrio*	1216	1099	1047	0.0013	0.0011	0.0011
Otu22	*Dysgonomonas*	320	1737	1113	0.0003	0.0018	0.0012
Otu23	*Lactococcus*	3661	0	0	0.0038	0.0000	0.0000
Otu26	*Orbus*	4143	16988	14840	0.0043	0.0176	0.0156
Otu280	*Klebsiella*	2612	801	2703	0.0027	0.0008	0.0028
Otu281	*Klebsiella*	2380	499	699	0.0025	0.0005	0.0007
Otu29	*Citrobacter*	23809	29274	22734	0.0249	0.0304	0.0238
Otu3	*Vagococcus*	87985	78421	25611	0.0921	0.0815	0.0269
Otu302	*Empedobacter*	1760	782	649	0.0018	0.0008	0.0007
Otu319	*Escherichia*	1121	743	1272	0.0012	0.0008	0.0013
Otu320	*Citrobacter*	2423	1598	1077	0.0025	0.0017	0.0011

Otu33	*Enterobacter*	11201	18300	12627	0.0117	0.0190	0.0132
Otu379	*Enterobacter*	2518	1077	7447	0.0026	0.0011	0.0078
Otu4	Enterobacteriaceae;Ambiguous_taxa	92609	149446	115852	0.0970	0.1552	0.1215
Otu40	*Orbus*	3701	3544	4810	0.0039	0.0037	0.0050
Otu45	*Klebsiella*	2047	422	1223	0.0021	0.0004	0.0013
Otu47	*Klebsiella*	4484	8769	9433	0.0047	0.0091	0.0099
Otu488	*Klebsiella*	1051	1081	1804	0.0011	0.0011	0.0019
Otu49	*Desulfovibrio*	279	1464	2658	0.0003	0.0015	0.0028
Otu5	*Providencia*	67508	31351	17249	0.0707	0.0326	0.0181
Otu52	*Shimwellia*	1333	946	1304	0.0014	0.0010	0.0014
Otu53	*Orbus*	3155	1089	1647	0.0033	0.0011	0.0017
Otu59	*Desulfovibrio*	61	4471	7183	0.0001	0.0046	0.0075
Otu6	*Desulfovibrio*	902	19174	45848	0.0009	0.0199	0.0481
Otu7	*Citrobacter*	58784	122492	153589	0.0616	0.1272	0.1610
Otu70	*Klebsiella*	21603	18064	32968	0.0226	0.0188	0.0346
Otu72	*Klebsiella*	5830	18748	9983	0.0061	0.0195	0.0105
Otu8	*Orbus*	58612	14849	10526	0.0614	0.0154	0.0110
Otu9	*Dysgonomonas*	2034	30924	5498	0.0021	0.0321	0.0058
Otu97	*Empedobacter*	2365	1134	1612	0.0025	0.0012	0.0017
	**Sum per area**	954886	962806	953800			
	**Total Reads**		2871492				

Supplementary Table S2: Nineteen OTUs remained unassigned and were classified at lower similarities. These unassigned OTUs could potentially belong to new uncharacterized taxa. The column titled "Taxonomy assigned at" contains the % similarity threshold that each sequence was classified during taxonomy assignment.

OTU	Identified as	Taxonomy assigned at
Otu1	Dysgonomonadaceae; *Dysgonomonas*	90 (potential new genus)
Otu10	Orbaceae; *Candidatus Schmidhempelia*	94 (potential new species)
Otu14	Orbaceae; *Orbus*	94 (potential new species)
Otu15	Dysgonomonadaceae; *Dysgonomonas*	94 (potential new species)
Otu18	Halomonadaceae; *Carnimonas*	94 (potential new species)
Otu21	Desulfovibrionaceae; *Desulfovibrio*	90 (potential new genus)
Otu22	Dysgonomonadaceae; *Dysgonomonas*	94 (potential new species)
Otu40	Orbaceae; *Orbus*	94 (potential new species)
Otu49	Desulfovibrionaceae; *Desulfovibrio*	90 (potential new genus)
Otu52	Enterobacteriaceae; *Shimwellia*	94 (potential new species)

Otu53	Orbaceae; *Orbus*	94 (potential new species)
Otu59	Desulfovibrionaceae; *Desulfovibrio*	94 (potential new species)
Otu97	Weeksellaceae; *Empedobacter*;	81 (potential new family)
Otu119	Orbaceae; *Orbus*	90 (potential new genus)
Otu123	Enterobacteriaceae; *Enterobacter*	90 (potential new genus)
Otu200	Orbaceae; *Candidatus* Schmidhempelia	94 (potential new species)
Otu302	Weeksellaceae; *Empedobacter*	81 (potential new family)
Otu319	Enterobacteriaceae; *Escherichia-Shigella*	94 (potential new species)
Otu488	Enterobacteriaceae; *Klebsiella*	94 (potential new species)

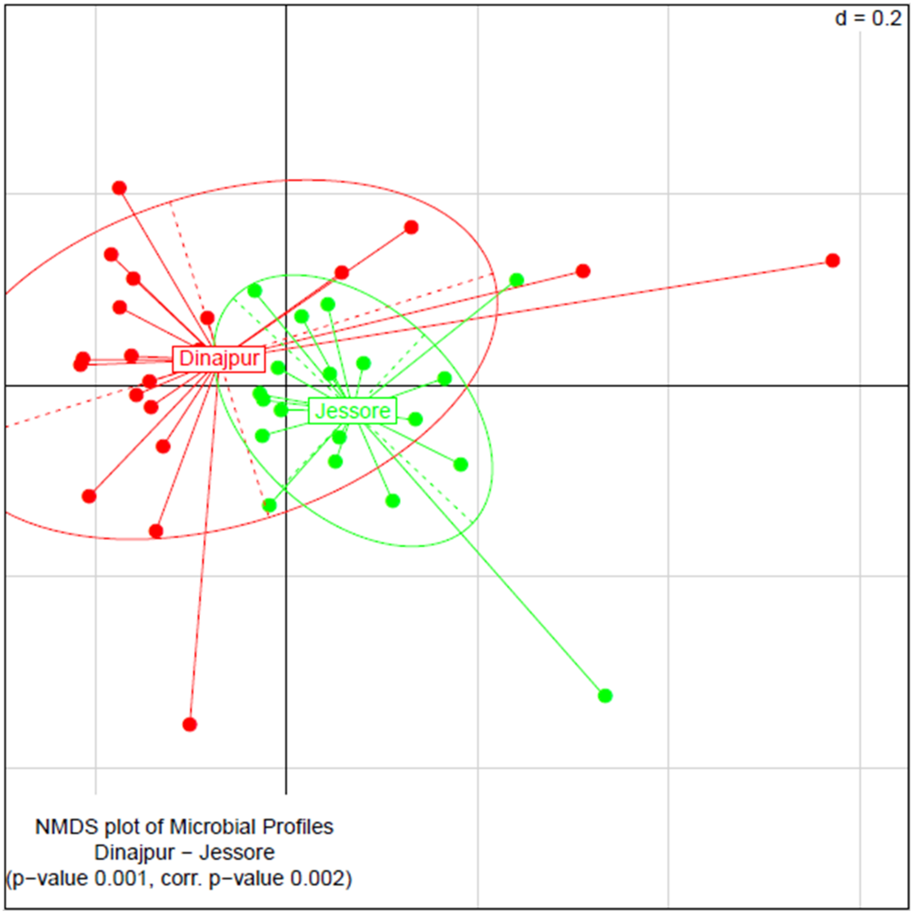

NMDS plot of Microbial Profiles
Dinajpur − Jessore
(p−value 0.001, corr. p−value 0.002)

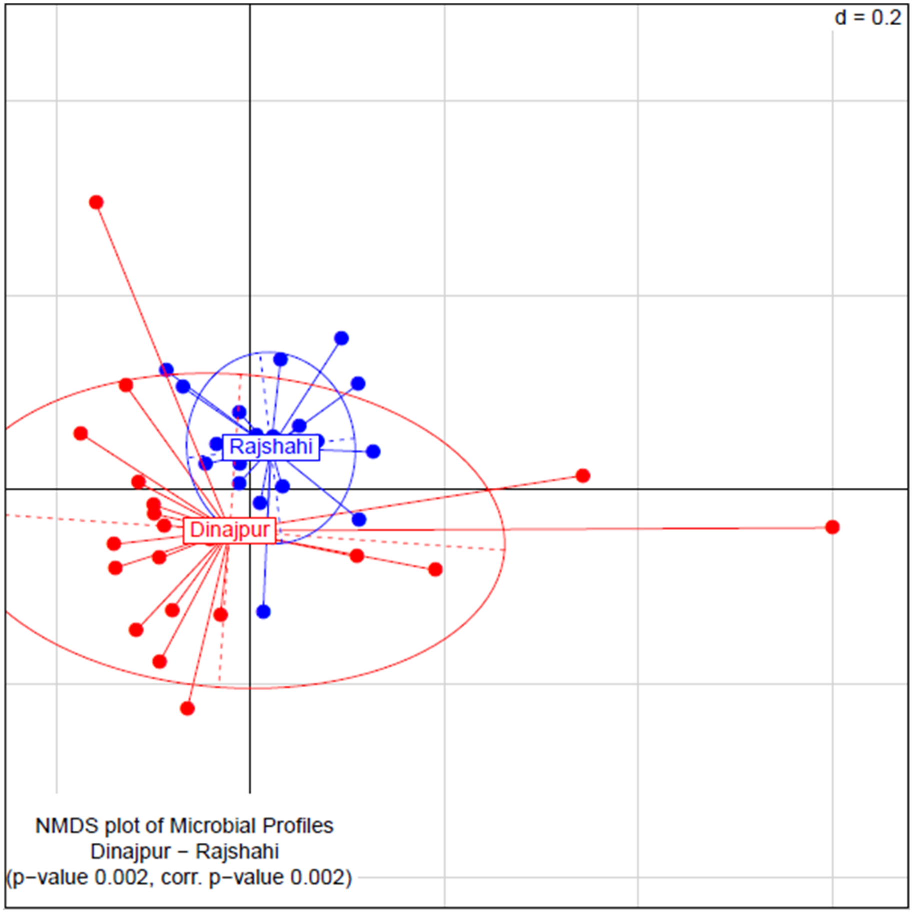
d = 0.2
Rajshahi
Dinajpur
NMDS plot of Microbial Profiles
Dinajpur − Rajshahi
(p−value 0.002, corr. p−value 0.002)

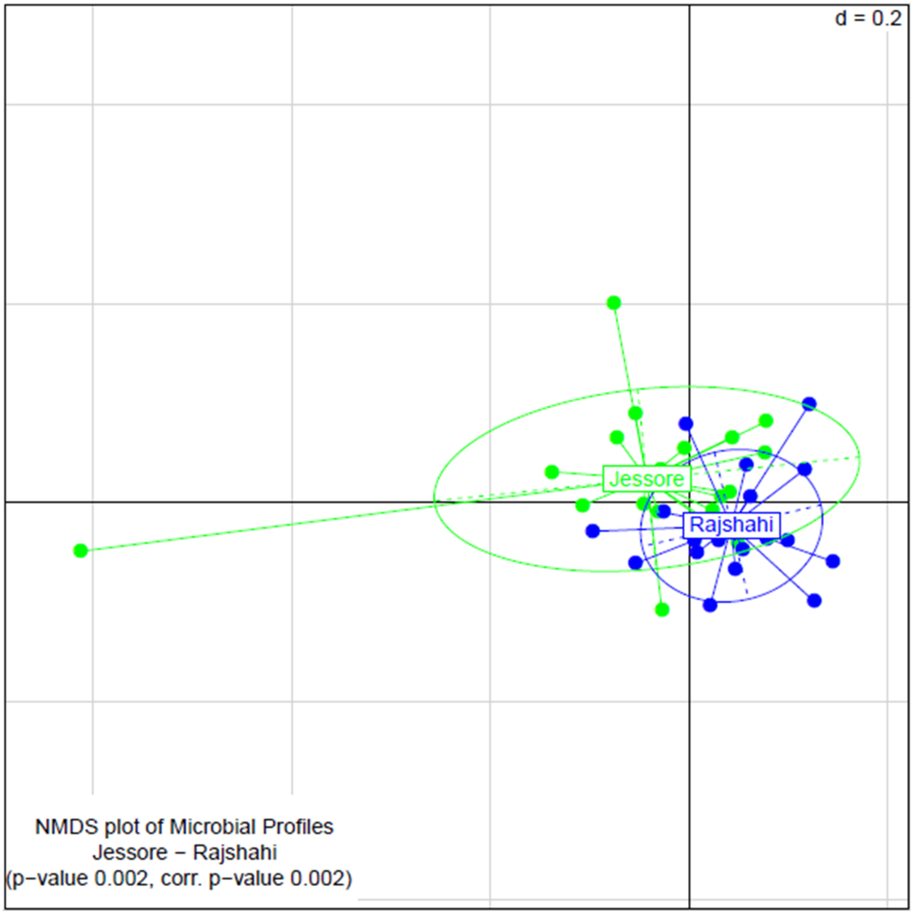

Supplementary Figure S1: Pairwise non-metric multidimensional scaling (NMDS) plot of bacterial communities for *Z. cucurbitae* samples collected from Dinajpur (red), Jessore (green) and Rajshahi (blue). All p-values are below 0.05 indicating statistically significant differences. 'd' indicates dissimilarity scale of the grid (d=0.2 mean that the distance between two grid lines represents approximately 20% dissimilarity between the samples).

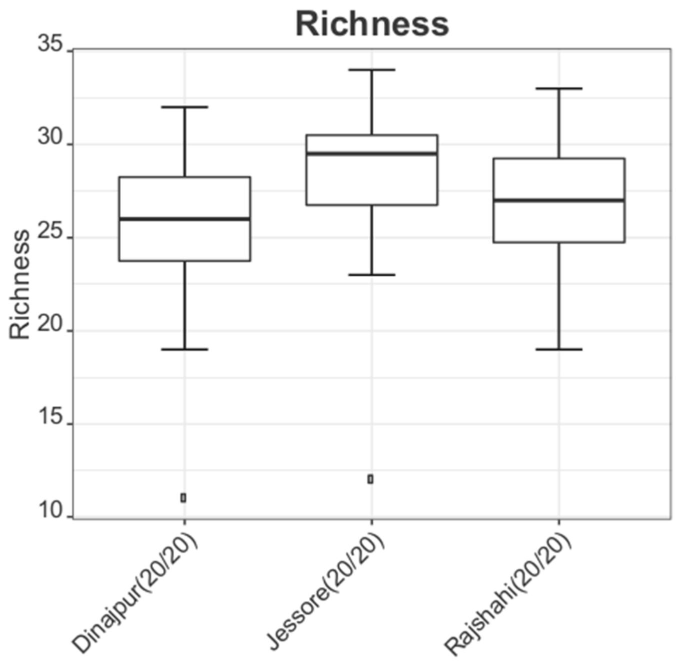

Kruskal−Wallis Rank Sum Test − all groups

	p−value	Adj. p−value
Richness	0.0487	0.138

Wilcoxon Rank Sum Test − pairwise

Groups	p−value	Adj. p−value
1−2	0.0166	0.0498

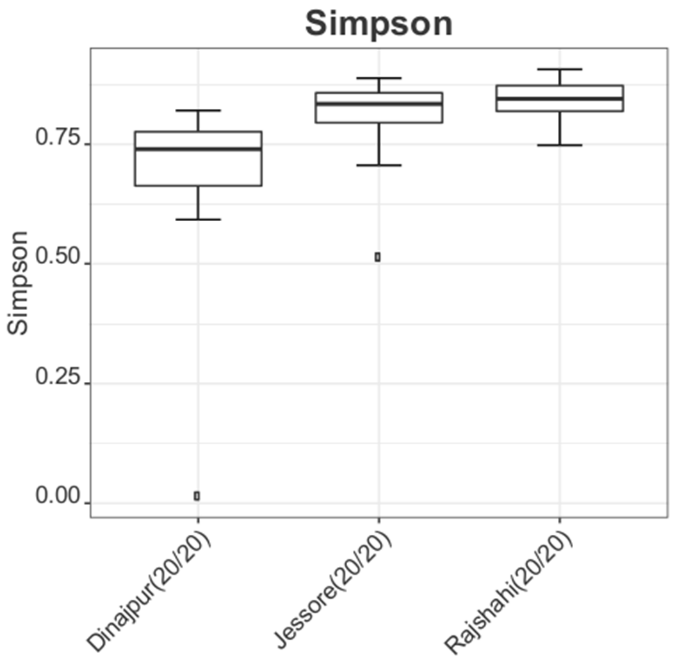

Kruskal−Wallis Rank Sum Test − all groups

	p−value	Adj. p−value
Simpson	0	0

Wilcoxon Rank Sum Test − pairwise

Groups	p−value	Adj. p−value
1−2	0	0
1−3	0	0

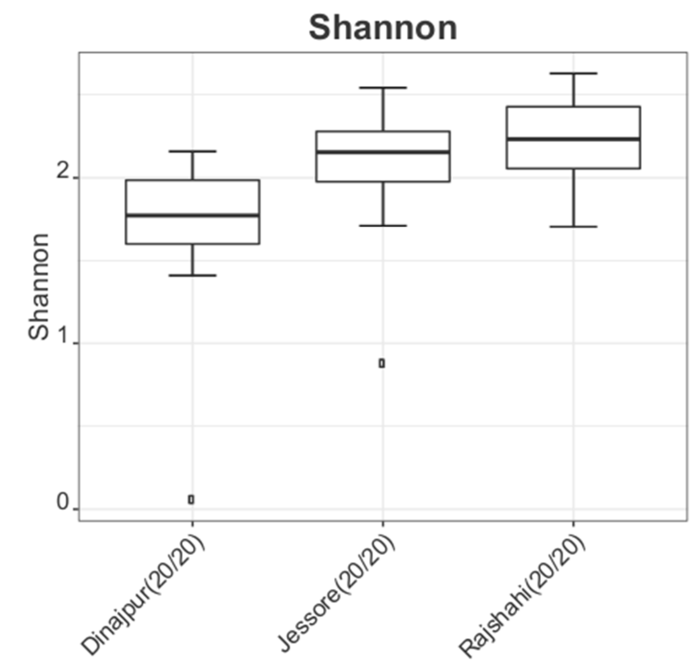

Kruskal−Wallis Rank Sum Test − all groups

	p−value	Adj. p−value
Shannon	0	0

Wilcoxon Rank Sum Test − pairwise

Groups	p−value	Adj. p−value
1−2	2e−04	3e−04
1−3	0e+00	0e+00

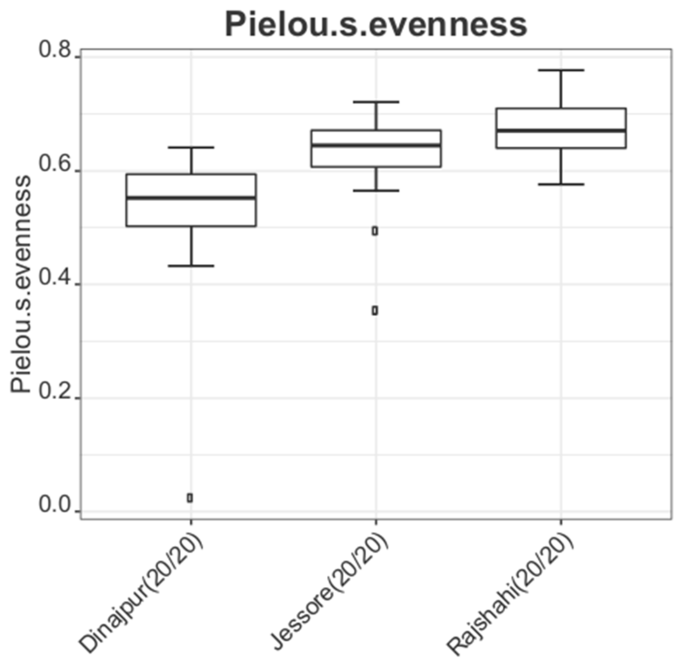

Kruskal−Wallis Rank Sum Test − all groups

	p−value	Adj. p−value
Pielou.s.evenness	0	0

Wilcoxon Rank Sum Test − pairwise

Groups	p−value	Adj. p−value
1−2	0.0001	0.0002
1−3	0.0000	0.0000
2−3	0.0491	0.0491

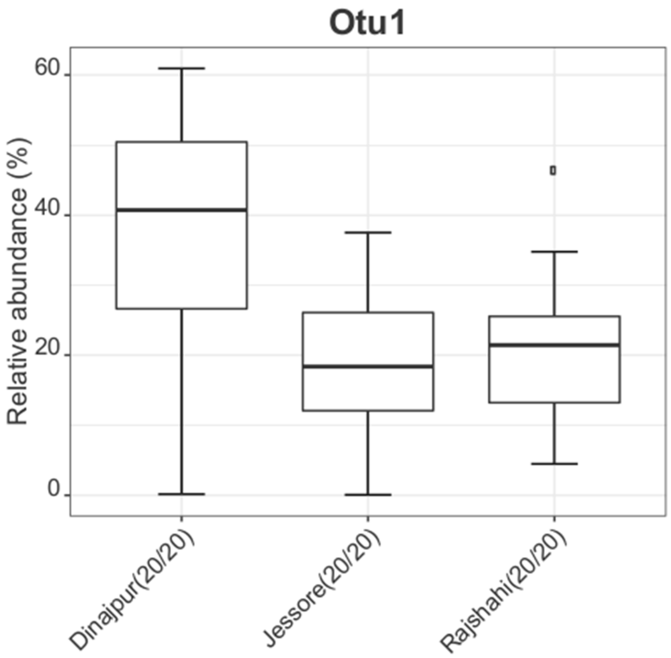

ruskal−Wallis Rank Sum Test − all group:

	p−value	Adj. p−value
Otu1	0.0011	0.0094

Wilcoxon Rank Sum Test − pairwise

Groups	p−value	Adj. p−value
1−2	6e−04	0.0018
1−3	2e−03	0.0030

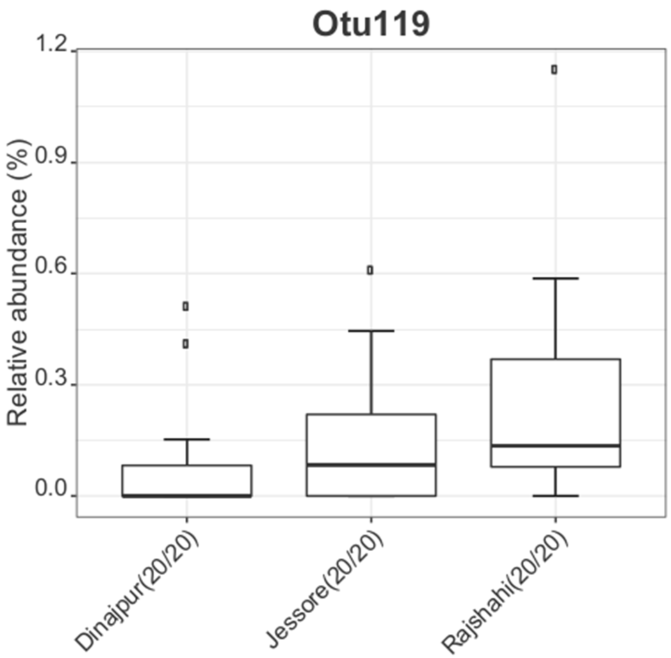

Kruskal−Wallis Rank Sum Test − all groups

	p−value	Adj. p−value
Otu119	0.0283	0.0902

Wilcoxon Rank Sum Test − pairwise

Groups	p−value	Adj. p−value
1−3	0.0086	0.0258

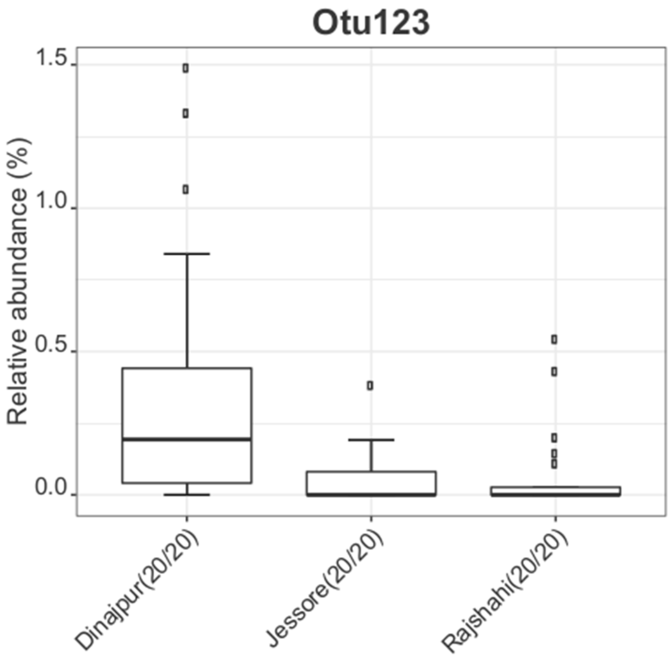

Kruskal−Wallis Rank Sum Test − all groups

	p−value	Adj. p−value
Otu123	6e−04	0.0061

Wilcoxon Rank Sum Test − pairwise

Groups	p−value	Adj. p−value
1−2	0.001	0.003
1−3	0.002	0.003

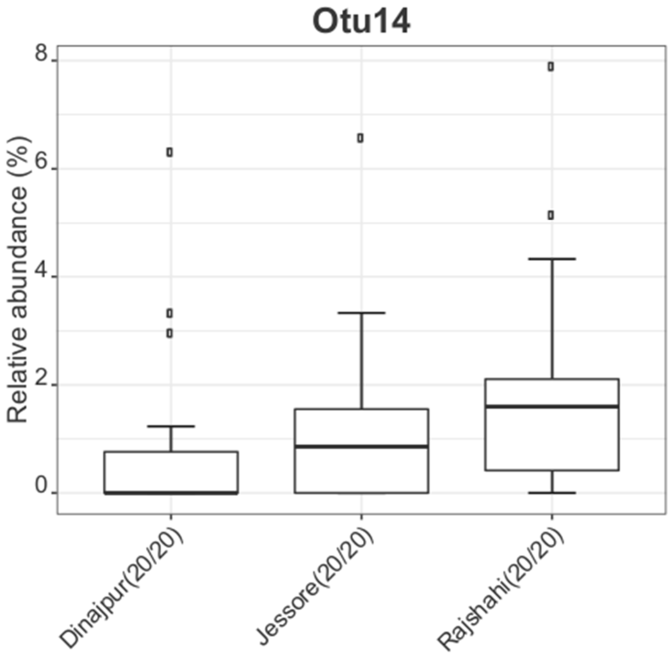

Kruskal−Wallis Rank Sum Test − all groups

	p−value	Adj. p−value
Otu14	0.0261	0.0902

Wilcoxon Rank Sum Test − pairwise

Groups	p−value	Adj. p−value
1−3	0.0107	0.0321

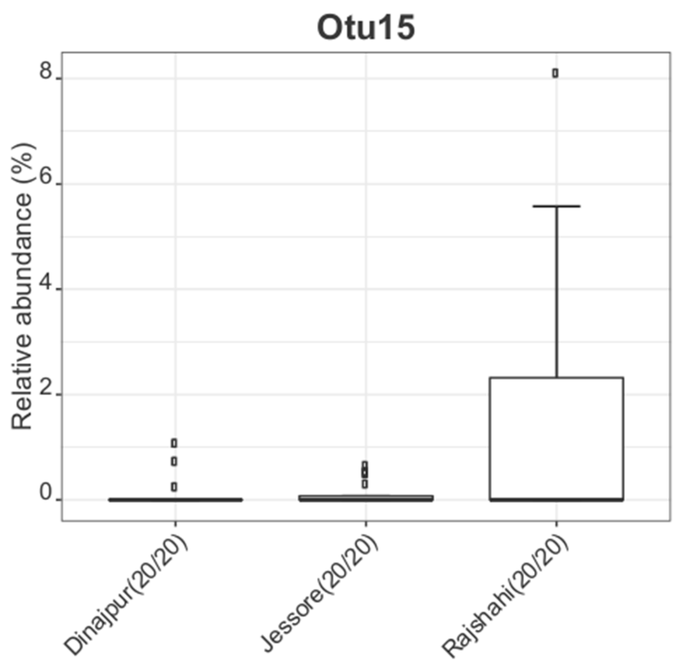

Kruskal−Wallis Rank Sum Test − all groups

	p−value	Adj. p−value
Otu15	0.034	0.102

Wilcoxon Rank Sum Test − pairwise

Groups	p−value	Adj. p−value
1−3	0.0195	0.0585

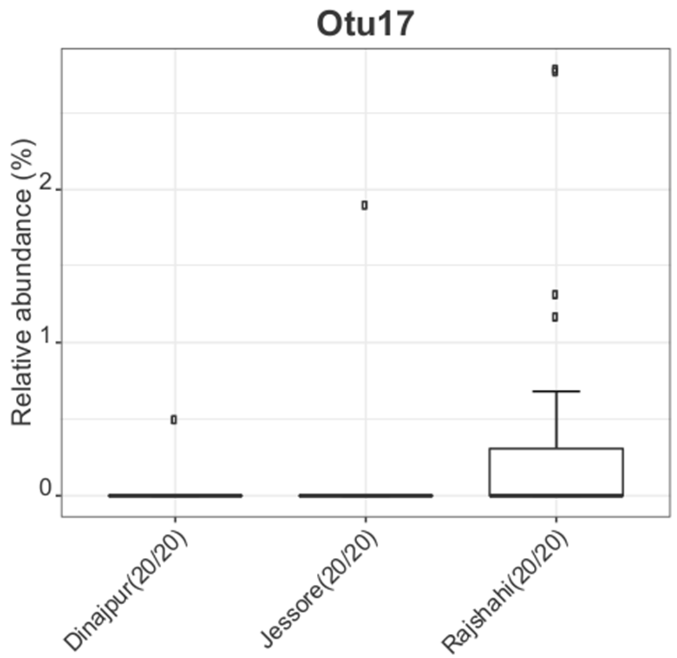

Kruskal−Wallis Rank Sum Test − all groups

	p−value	Adj. p−value
Otu17	0.0275	0.0902

Wilcoxon Rank Sum Test − pairwise

Groups	p−value	Adj. p−value
1−3	0.0354	0.0714
2−3	0.0476	0.0714

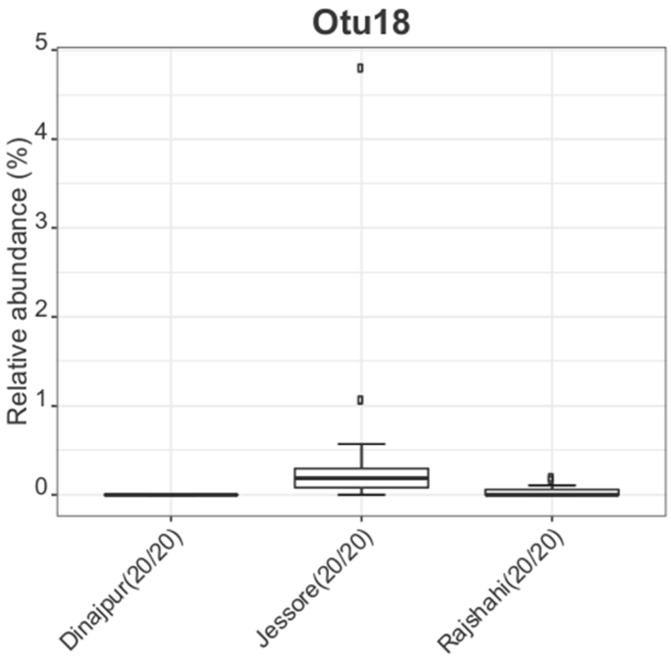

Kruskal-Wallis Rank Sum Test − all groups

	p−value	Adj. p−value
Otu18	0	0

Wilcoxon Rank Sum Test − pairwise

Groups	p−value	Adj. p−value
1−2	0.0000	0.0000
1−3	0.0045	0.0045
2−3	0.0001	0.0002

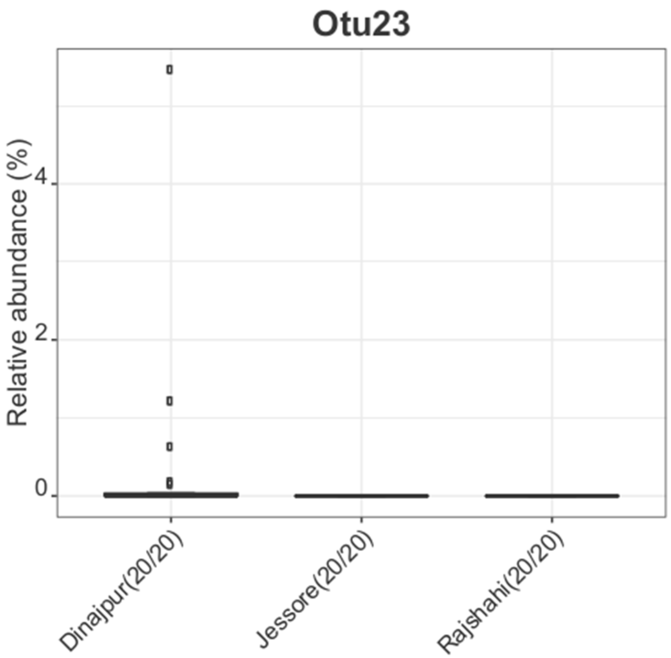

Kruskal-Wallis Rank Sum Test − all groups

	p−value	Adj. p−value
Otu23	0.0047	0.024

Wilcoxon Rank Sum Test − pairwise

Groups	p−value	Adj. p−value
1−2	0.0198	0.0198
1−3	0.0198	0.0198

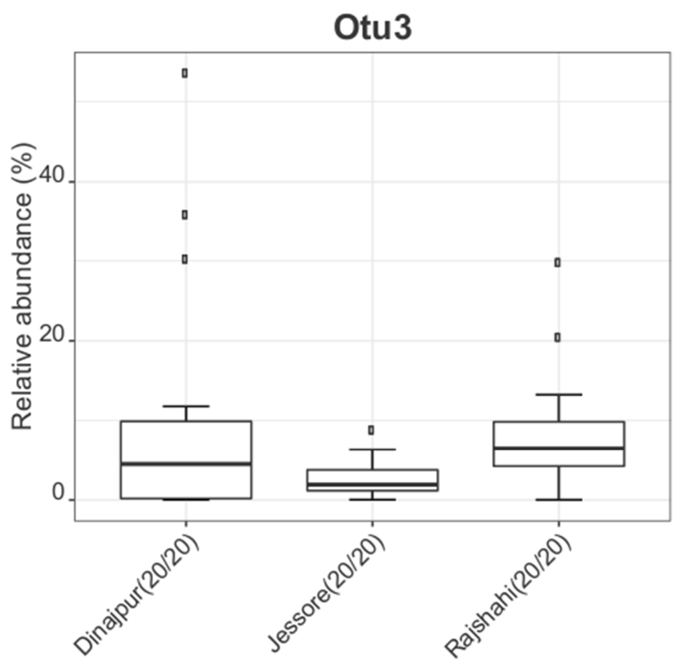

ruskal−Wallis Rank Sum Test − all group

	p−value	Adj. p−value
Otu3	0.0179	0.0702

Wilcoxon Rank Sum Test − pairwise

Groups	p−value	Adj. p−value
2−3	4e−04	0.0012

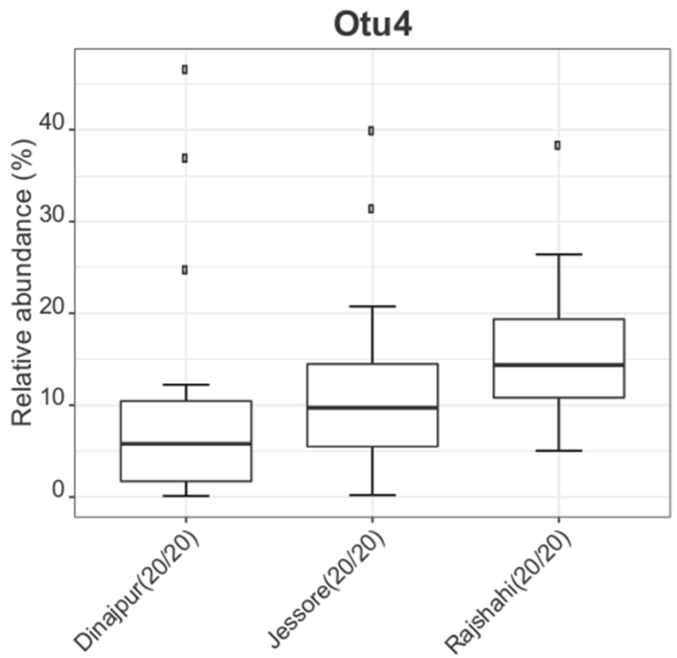

ruskal−Wallis Rank Sum Test − all group

	p−value	Adj. p−value
Otu4	0.0032	0.0204

Wilcoxon Rank Sum Test − pairwise

Groups	p−value	Adj. p−value
1−3	0.0009	0.0027
2−3	0.0402	0.0603

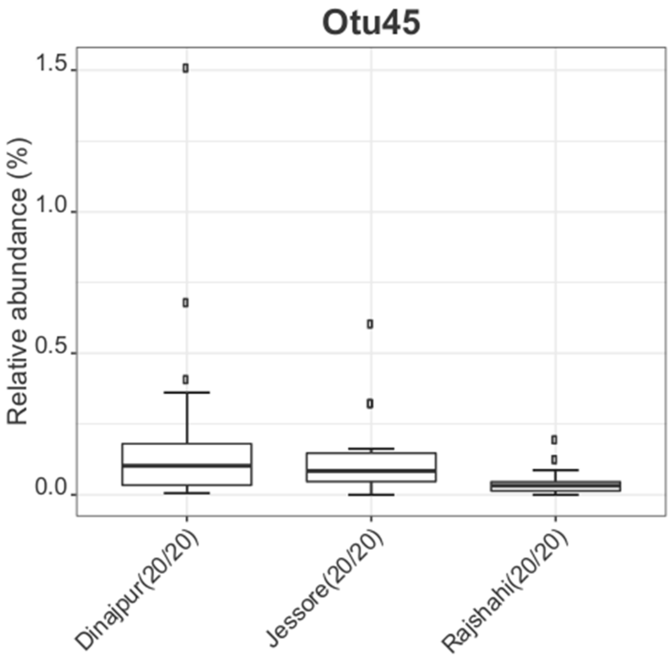

Kruskal−Wallis Rank Sum Test − all groups

	p−value	Adj. p−value
Otu45	0.0082	0.038

Wilcoxon Rank Sum Test − pairwise

Groups	p−value	Adj. p−value
1−3	0.0095	0.0142
2−3	0.0058	0.0142

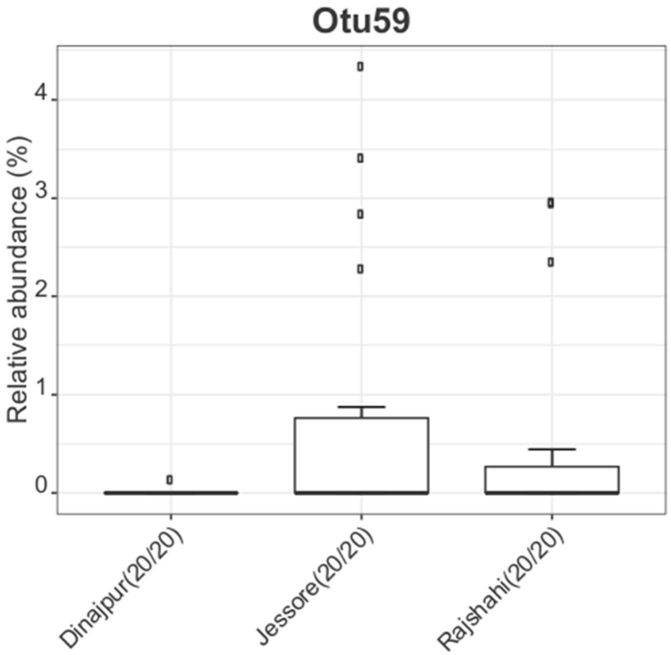

Kruskal−Wallis Rank Sum Test − all groups

	p−value	Adj. p−value
Otu59	0.0128	0.0544

Wilcoxon Rank Sum Test − pairwise

Groups	p−value	Adj. p−value
1−2	0.0028	0.0084
1−3	0.0320	0.0480

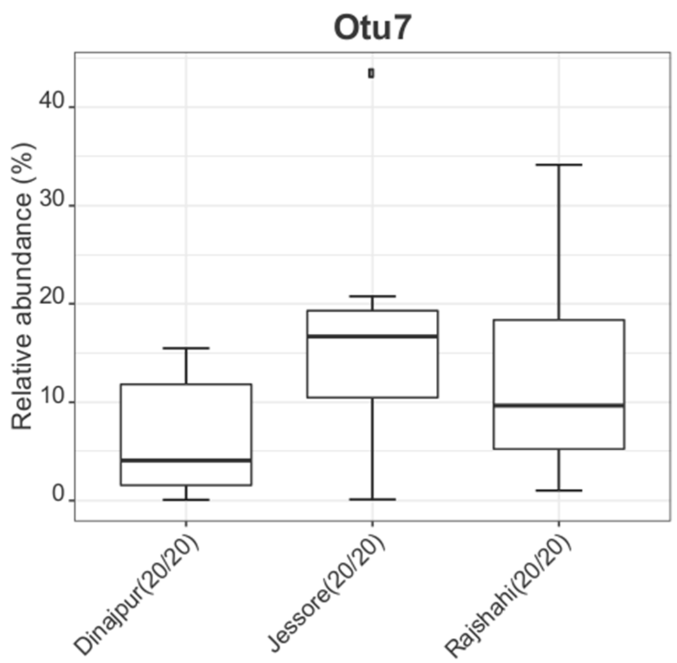

ruskal−Wallis Rank Sum Test − all group:

	p−value	Adj. p−value
Otu7	0.002	0.0146

Wilcoxon Rank Sum Test − pairwise

Groups	p−value	Adj. p−value
1−2	0.0004	0.0012
1−3	0.0155	0.0232

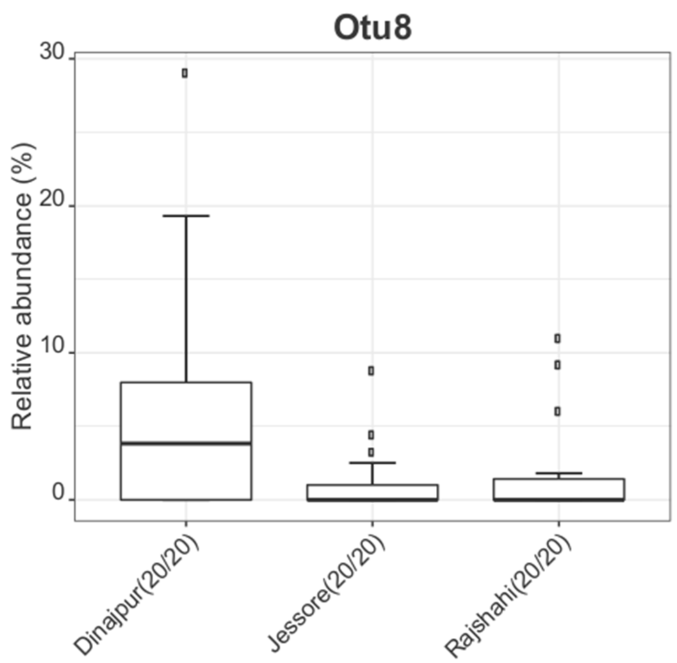

ruskal−Wallis Rank Sum Test − all group:

	p−value	Adj. p−value
Otu8	0.0046	0.024

Wilcoxon Rank Sum Test − pairwise

Groups	p−value	Adj. p−value
1−2	0.0044	0.0132
1−3	0.0089	0.0134

Supplementary Figure S2: Alpha diversity indices and pairwise comparisons of OTUs that show statistically significant differences (p-value<0.05) among the three tested areas.

Dinajpur Network (Genus level)

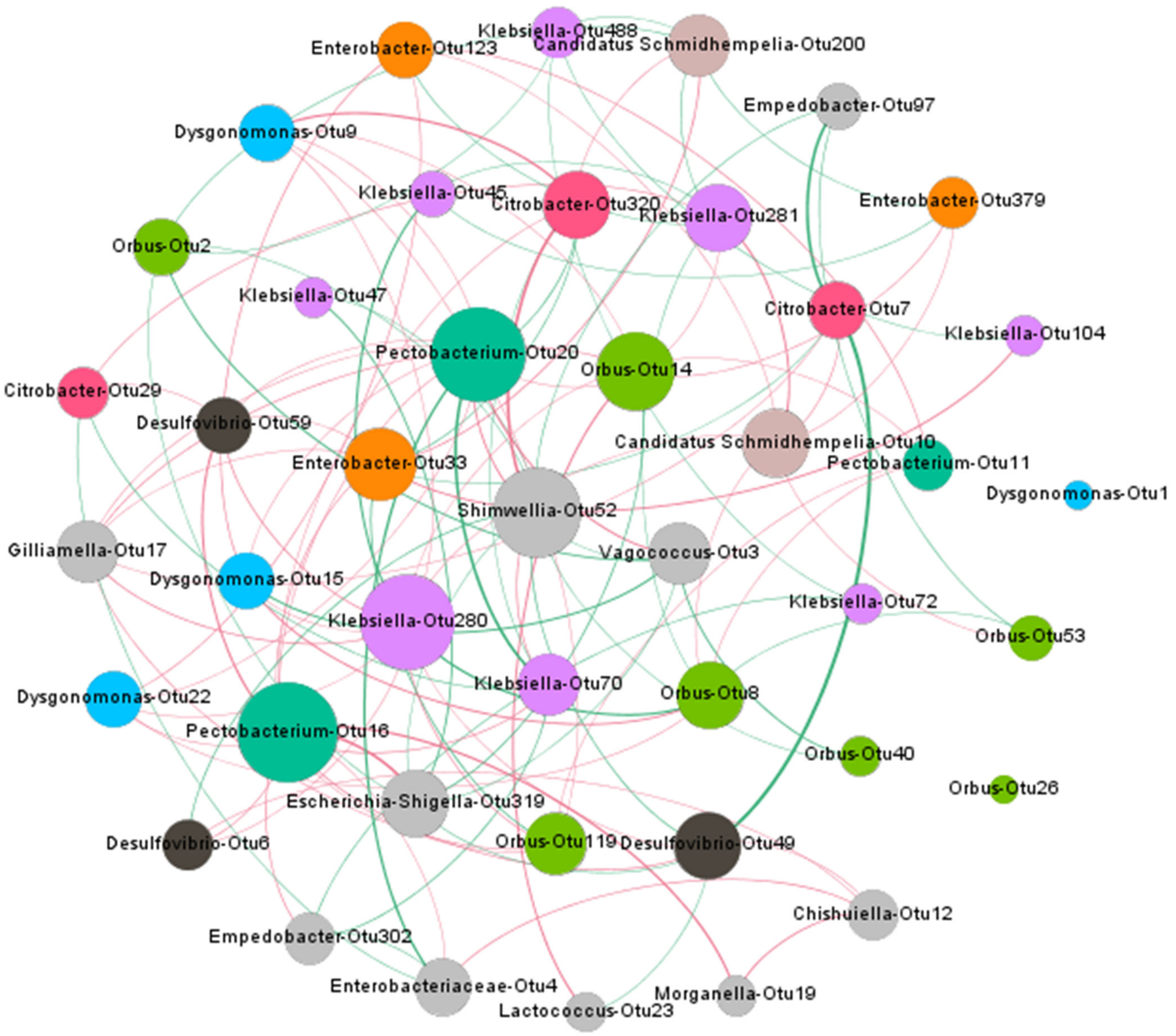

Jessore Network (Genus level)

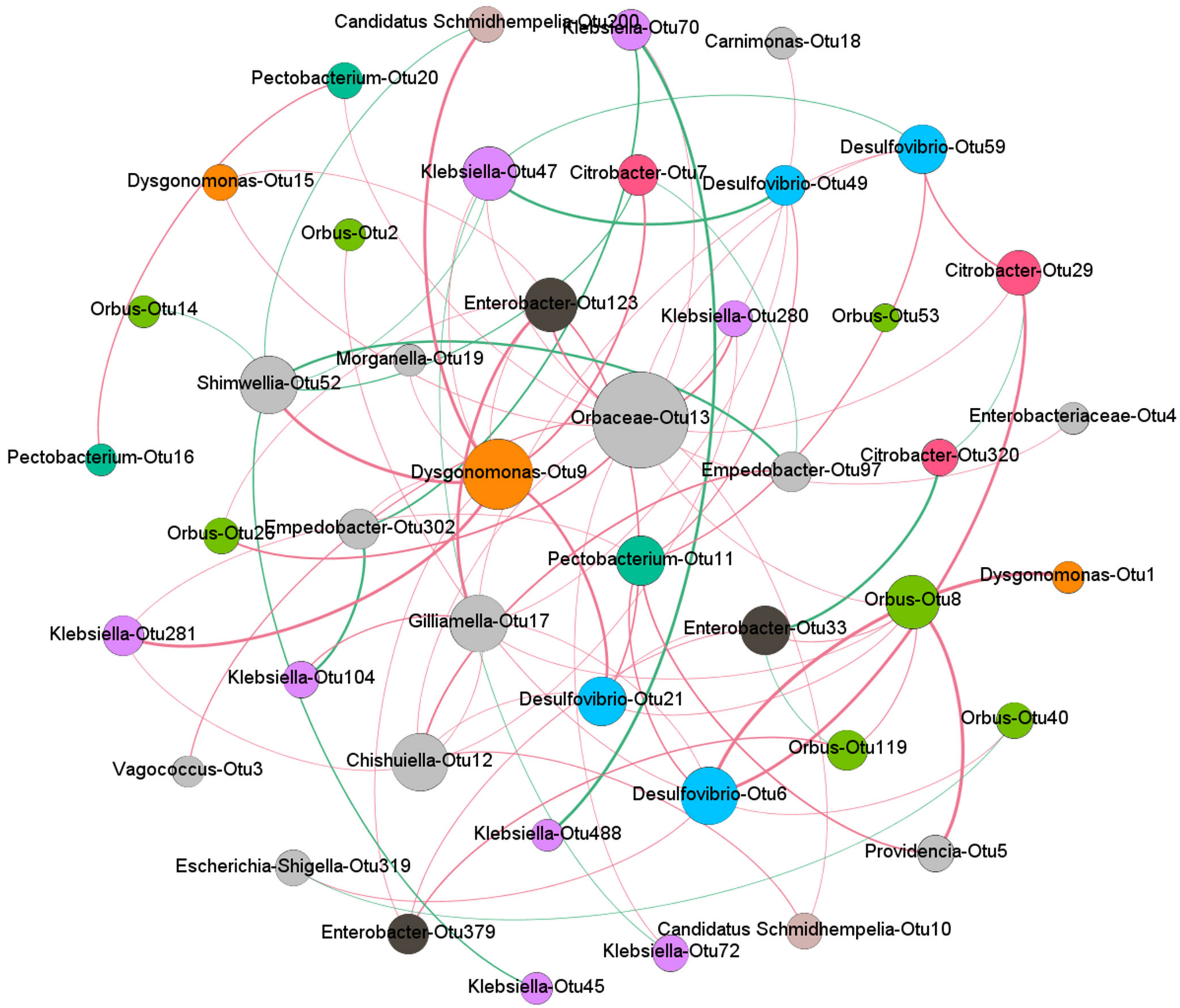

Rajshahi Network (Genus level)

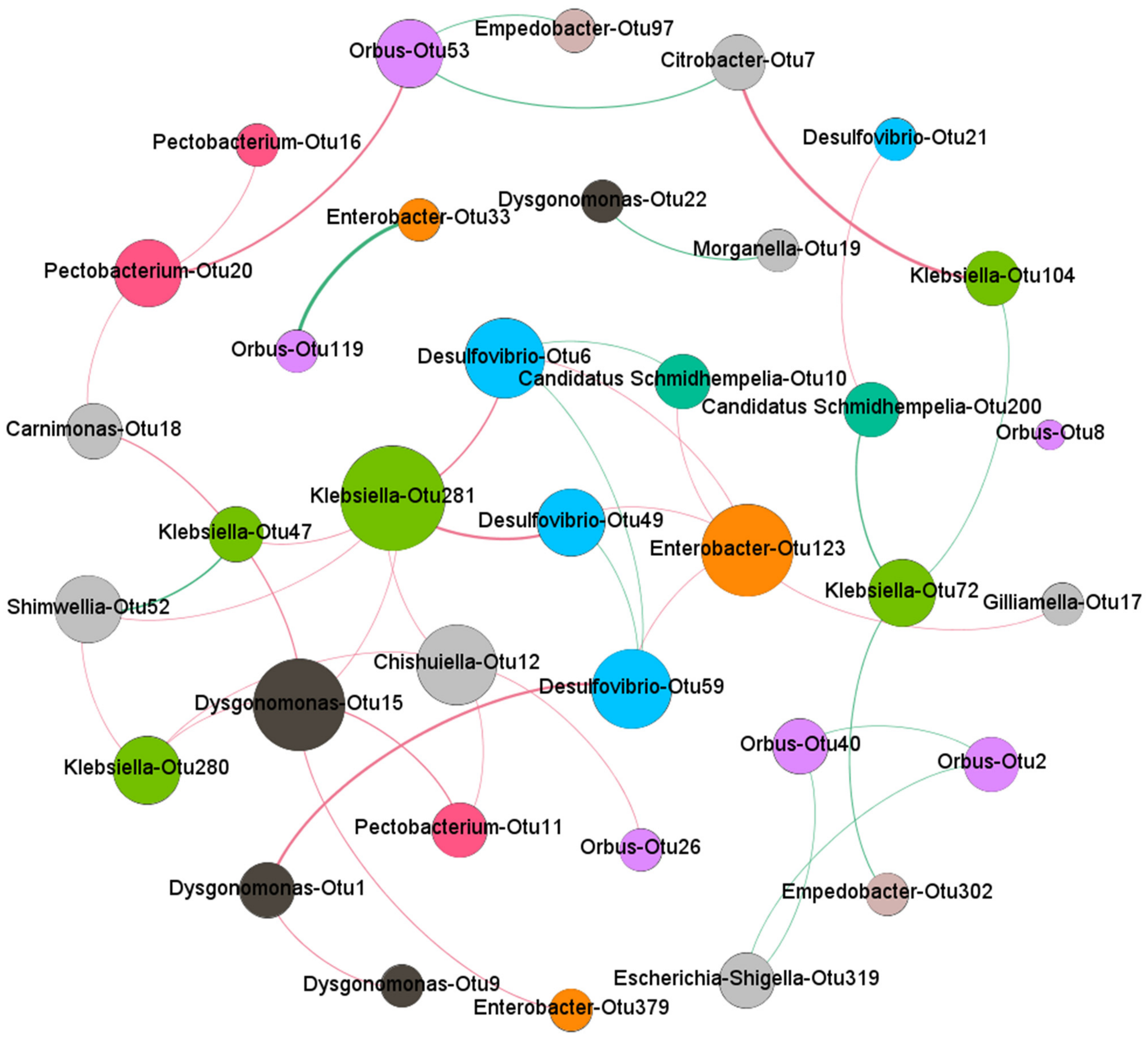

Supplementary Figure S3: Mutual exclusion and co-occurrence networks among OTUs (genera) in the three tested areas.

Supplementary Table S3: Network statistics for each region

	Nodes	Edges	Average Clustering Coefficient:	Total triangles	Mutual exclusion %	Copresence %	Mutual exclusion (number of edges)	Copresence (number of edges)
**Dinajpur**	43	113	0.205	42	59.29	40.71	67	46
**Jessore**	45	77	0.349	16	77.92	22.08	60	17
**Rajshahi**	36	40	0.291	5	65	35	26	14

Supplementary Table S4: Network statistics for the OTUs in the three tested areas. The column titled "degree" contains the total number of interactions. The column titled "posdegree" contains the number of positive interactions, and "negdegree" negative interactions.

Network Statistics Dinajpur

Label	lineage	posdegree	abundance	degree	negdegree	sample count	clustering	triangles
Pectobacterium-Otu16	Bacteria_Proteobacteria_Gammaproteobacteria_Enterobacteriales_Enterobacteriaceae_Pectobacterium_OTU_Otu16	4	0.040962	25	21	5	0.153846	12
Pectobacterium-Otu20	Bacteria_Proteobacteria_Gammaproteobacteria_Enterobacteriales_Enterobacteriaceae_Pectobacterium_OTU_Otu20	5	0.096059	24	19	10	0.19697	13
Shimwellia-Otu52	Bacteria_Proteobacteria_Gammaproteobacteria_Enterobacteriales_Enterobacteriaceae_Shimwellia_OTU_Otu52	19	0.027907	22	3	12	0.181818	10
Gilliamella-Otu17	Bacteria_Proteobacteria_Gammaproteobacteria_Orbales_Orbaceae_Gilliamella_OTU_Otu17	5	0.004948	18	13	1	0.333333	5
Escherichia-Shigella-Otu319	Bacteria_Proteobacteria_Gammaproteobacteria_Enterobacteriales_Enterobacteriaceae_Escherichia-Shigella_OTU_Otu319	17	0.023458	18	1	18	0.285714	6
Orbus-Otu14	Bacteria_Proteobacteria_Gammaproteobacteria_Orbales_Orbaceae_Orbus_OTU_Otu14	4	0.158718	16	12	7	0.111111	4
Klebsiella-Otu280	Bacteria_Proteobacteria_Gammaproteobacteria_Enterobacteriales_Enterobacteriaceae_Klebsiella_OTU_Otu280	5	0.054844	16	11	8	0.106061	7
Candidatus Schmidhempelia-Otu10	Bacteria_Proteobacteria_Gammaproteobacteria_Orbales_Orbaceae_Candidatus Schmidhempelia_OTU_Otu10	3	0.24238	14	11	3	0.190476	4
Citrobacter-Otu320	Bacteria_Proteobacteria_Gammaproteobacteria_Enterobacteriales_Enterobacteriaceae_Citrobacter_OTU_Otu320	7	0.050372	13	6	16	0.380952	8
Desulfovibrio-Otu49	Bacteria_Proteobacteria_Deltaproteobacteria_Desulfovibrionales_Desulfovibrionaceae_Desulfovibrio_OTU_Otu49	9	0.005797	13	4	3	0.095238	2

Dysgonomonas-Otu9	Bacteria_Bacteroidetes_Bacteroidia_Bacteroidales_Dysgonomonadaceae_Dysgonomonas_OTU_Otu9	3	0.043678	13	10	2	0.4	4
Orbus-Otu8	Bacteria_Proteobacteria_Gammaproteobacteria_Orbales_Orbaceae_Orbus_OTU_Otu8	5	1.228702	13	8	14	0.238095	5
Desulfovibrio-Otu59	Bacteria_Proteobacteria_Deltaproteobacteria_Desulfovibrionales_Desulfovibrionaceae_Desulfovibrio_OTU_Otu59	4	0.001294	13	9	1	0.2	2
Orbus-Otu2	Bacteria_Proteobacteria_Gammaproteobacteria_Orbales_Orbaceae_Orbus_OTU_Otu2	10	1.830101	12	2	20	0.4	4
Orbus-Otu40	Bacteria_Proteobacteria_Gammaproteobacteria_Orbales_Orbaceae_Orbus_OTU_Otu40	11	0.077653	11	0	20	1	1
Enterobacter-Otu33	Bacteria_Proteobacteria_Gammaproteobacteria_Enterobacteriales_Enterobacteriaceae_Enterobacter_OTU_Otu33	3	0.23315	11	8	11	0.214286	6
Dysgonomonas-Otu15	Bacteria_Bacteroidetes_Bacteroidia_Bacteroidales_Dysgonomonadaceae_Dysgonomonas_OTU_Otu15	6	0.020292	10	4	3	0.2	2
Enterobacter-Otu123	Bacteria_Proteobacteria_Gammaproteobacteria_Enterobacteriales_Enterobacteriaceae_Enterobacter_OTU_Otu123	2	0.075358	10	8	15	0.1	1
Vagococcus-Otu3	Bacteria_Firmicutes_Bacilli_Lactobacillales_Enterococcaceae_Vagococcus_OTU_Otu3	8	1.85056	10	2	20	0.4	6
Klebsiella-Otu47	Bacteria_Proteobacteria_Gammaproteobacteria_Enterobacteriales_Enterobacteriaceae_Klebsiella_OTU_Otu47	9	0.093839	9	0	20	1	1
Candidatus Schmidhempelia-Otu200	Bacteria_Proteobacteria_Gammaproteobacteria_Orbales_Orbaceae_Candidatus Schmidhempelia_OTU_Otu200	5	0.04526	9	4	14	0.133333	2
Klebsiella-Otu281	Bacteria_Proteobacteria_Gammaproteobacteria_Enterobacteriales_Enterobacteriaceae_Klebsiella_OTU_Otu281	6	0.050047	9	3	6	0.095238	2
Dysgonomonas-Otu22	Bacteria_Bacteroidetes_Bacteroidia_Bacteroidales_Dysgonomonadaceae_Dysgonomonas_OTU_Otu22	2	0.00677	9	7	3	0.2	2
Enterobacteriaceae-Otu4	Bacteria_Proteobacteria_Gammaproteobacteria_Enterobacteriales_Enterobacteriaceae_Ambiguous_taxa_OTU_Otu4	6	1.929522	9	3	20	0.2	2
Klebsiella-Otu488	Bacteria_Proteobacteria_Gammaproteobacteria_Enterobacteriales_Enterobacteriaceae_Klebsiella_OTU_Otu488	8	0.022098	8	0	16	0.166667	1
Klebsiella-Otu70	Bacteria_Proteobacteria_Gammaproteobacteria_Enterobacteriales_Enterobacteriaceae_Klebsiella_OTU_Otu70	6	0.450208	8	2	20	0.4	6
Orbus-Otu119	Bacteria_Proteobacteria_Gammaproteobacteria_Orbales_Orbaceae_Orbus_OTU_Otu119	1	0.01588	8	7	9	0.266667	4
Citrobacter-Otu29	Bacteria_Proteobacteria_Gammaproteobacteria_Enterobacteriales_Enterobacteriaceae_Citrobacter_OTU_Otu29	5	0.49757	7	2	11	0	0
Desulfovibrio-Otu6	Bacteria_Proteobacteria_Deltaproteobacteria_Desulfovibrionales_Desulfovibrionaceae_Desulfovibrio_OTU_Otu6	3	0.018721	7	4	2	0	0

Morganella-Otu19	Bacteria_Proteobacteria_Gammaproteobacteria_Enterobacteriales_Enterobacteriaceae_Morganella_OTU_Otu19	0	0.004974	7	7	3	0	0
Citrobacter-Otu7	Bacteria_Proteobacteria_Gammaproteobacteria_Enterobacteriales_Enterobacteriaceae_Citrobacter_OTU_Otu7	3	1.228639	6	3	20	0.1	1
Enterobacter-Otu379	Bacteria_Proteobacteria_Gammaproteobacteria_Enterobacteriales_Enterobacteriaceae_Enterobacter_OTU_Otu379	3	0.052771	6	3	5	0	0
Chishuiella-Otu12	Bacteria_Bacteroidetes_Bacteroidia_Flavobacteriales_Weeksellaceae_Chishuiella_OTU_Otu12	0	0.166955	6	6	5	0	0
Pectobacterium-Otu11	Bacteria_Proteobacteria_Gammaproteobacteria_Enterobacteriales_Enterobacteriaceae_Pectobacterium_OTU_Otu11	2	0.056274	6	4	3	0	0
Empedobacter-Otu302	Bacteria_Bacteroidetes_Bacteroidia_Flavobacteriales_Weeksellaceae_Empedobacter_OTU_Otu302	4	0.036775	5	1	19	0.333333	2
Klebsiella-Otu45	Bacteria_Proteobacteria_Gammaproteobacteria_Enterobacteriales_Enterobacteriaceae_Klebsiella_OTU_Otu45	5	0.042986	5	0	20	0	0
Dysgonomonas-Otu1	Bacteria_Bacteroidetes_Bacteroidia_Bacteroidales_Dysgonomonadaceae_Dysgonomonas_OTU_Otu1	0	7.339884	4	4	20	0	0
Klebsiella-Otu72	Bacteria_Proteobacteria_Gammaproteobacteria_Enterobacteriales_Enterobacteriaceae_Klebsiella_OTU_Otu72	4	0.121892	4	0	9	0	0
Klebsiella-Otu104	Bacteria_Proteobacteria_Gammaproteobacteria_Enterobacteriales_Enterobacteriaceae_Klebsiella_OTU_Otu104	3	0.05164	4	1	19	0	0
Empedobacter-Otu97	Bacteria_Bacteroidetes_Bacteroidia_Flavobacteriales_Weeksellaceae_Empedobacter_OTU_Otu97	4	0.049557	4	0	19	0	0
Orbus-Otu53	Bacteria_Proteobacteria_Gammaproteobacteria_Orbales_Orbaceae_Orbus_OTU_Otu53	2	0.066213	3	1	18	0.333333	1
Lactococcus-Otu23	Bacteria_Firmicutes_Bacilli_Lactobacillales_Streptococcaceae_Lactococcus_OTU_Otu23	1	0.076143	2	1	5	0	0
Orbus-Otu26	Bacteria_Proteobacteria_Gammaproteobacteria_Orbales_Orbaceae_Orbus_OTU_Otu26	1	0.087969	1	0	4	0	0

Network Statistics Jessore

**Label**	**lineage**	**posdegree**	**abundance**	**degree**	**negdegree**	**sample count**	**clustering**	**triangles**
Shimwellia-Otu52	Bacteria_Proteobacteria_Gammaproteobacteria_Enterobacteriales_Enterobacteriaceae_Shimwellia_OTU_Otu52	7	0.027386	10	3	14	0.190476	4
Klebsiella-Otu47	Bacteria_Proteobacteria_Gammaproteobacteria_Enterobacteriales_Enterobacteriaceae_Klebsiella_OTU_Otu47	5	0.197447	8	3	20	0.2	3
Klebsiella-Otu488	Bacteria_Proteobacteria_Gammaproteobacteria_Enterobacteriales_Enterobacteriaceae_Klebsiella_OTU_Otu488	4	0.037462	10	6	19	0	0

Empedobacter-Otu97	Bacteria_Bacteroidetes_Bacteroidia_Flavobacteriales_Weeksellaceae_Empedobacter_OTU_Otu97	4	0.033827	7	3	19	0.333333	1
Citrobacter-Otu320	Bacteria_Proteobacteria_Gammaproteobacteria_Enterobacteriales_Enterobacteriaceae_Citrobacter_OTU_Otu320	3	0.022682	9	6	18	0	0
Citrobacter-Otu7	Bacteria_Proteobacteria_Gammaproteobacteria_Enterobacteriales_Enterobacteriaceae_Citrobacter_OTU_Otu7	3	3.230727	8	5	20	0.666667	2
Desulfovibrio-Otu49	Bacteria_Proteobacteria_Deltaproteobacteria_Desulfovibrionales_Desulfovibrionaceae_Desulfovibrio_OTU_Otu49	3	0.055999	6	3	9	0	0
Candidatus Schmidhempelia-Otu200	Bacteria_Proteobacteria_Gammaproteobacteria_Orbales_Orbaceae_Candidatus Schmidhempelia_OTU_Otu200	3	0.033906	6	3	17	1	1
Empedobacter-Otu302	Bacteria_Bacteroidetes_Bacteroidia_Flavobacteriales_Weeksellaceae_Empedobacter_OTU_Otu302	3	0.013494	6	3	19	0	0
Klebsiella-Otu45	Bacteria_Proteobacteria_Gammaproteobacteria_Enterobacteriales_Enterobacteriaceae_Klebsiella_OTU_Otu45	3	0.025667	5	2	19	0	0
Enterobacter-Otu33	Bacteria_Proteobacteria_Gammaproteobacteria_Enterobacteriales_Enterobacteriaceae_Enterobacter_OTU_Otu33	2	0.264489	9	7	11	0.3	3
Desulfovibrio-Otu59	Bacteria_Proteobacteria_Deltaproteobacteria_Desulfovibrionales_Desulfovibrionaceae_Desulfovibrio_OTU_Otu59	2	0.149779	9	7	9	0.2	2
Orbus-Otu40	Bacteria_Proteobacteria_Gammaproteobacteria_Orbales_Orbaceae_Orbus_OTU_Otu40	2	0.100807	9	7	19	1	1
Klebsiella-Otu70	Bacteria_Proteobacteria_Gammaproteobacteria_Enterobacteriales_Enterobacteriaceae_Klebsiella_OTU_Otu70	2	0.681153	8	6	20	0	0
Orbus-Otu14	Bacteria_Proteobacteria_Gammaproteobacteria_Orbales_Orbaceae_Orbus_OTU_Otu14	2	0.259174	4	2	13	0	0
Desulfovibrio-Otu6	Bacteria_Proteobacteria_Deltaproteobacteria_Desulfovibrionales_Desulfovibrionaceae_Desulfovibrio_OTU_Otu6	1	0.988332	12	11	7	0.047619	1
Orbus-Otu8	Bacteria_Proteobacteria_Gammaproteobacteria_Orbales_Orbaceae_Orbus_OTU_Otu8	1	0.219271	11	10	7	0.133333	2
Orbus-Otu119	Bacteria_Proteobacteria_Gammaproteobacteria_Orbales_Orbaceae_Orbus_OTU_Otu119	1	0.030519	9	8	14	0.666667	2
Enterobacter-Otu123	Bacteria_Proteobacteria_Gammaproteobacteria_Enterobacteriales_Enterobacteriaceae_Enterobacter_OTU_Otu123	1	0.010486	9	8	6	0.2	3
Citrobacter-Otu29	Bacteria_Proteobacteria_Gammaproteobacteria_Enterobacteriales_Enterobacteriaceae_Citrobacter_OTU_Otu29	1	0.476239	9	8	11	0.166667	1
Escherichia-Shigella-Otu319	Bacteria_Proteobacteria_Gammaproteobacteria_Enterobacteriales_Enterobacteriaceae_Escherichia-Shigella_OTU_Otu319	1	0.026777	8	7	19	1	1
Klebsiella-Otu72	Bacteria_Proteobacteria_Gammaproteobacteria_Enterobacteriales_Enterobacteriaceae_Klebsiella_OTU_Otu72	1	0.208961	6	5	9	1	1

Klebsiella-Otu280	Bacteria_Proteobacteria_Gammaproteobacteria_Enterobacteriales_Enterobacteriaceae_Klebsiella_OTU_Otu280	1	0.056576	5	4	8	1	1
Klebsiella-Otu104	Bacteria_Proteobacteria_Gammaproteobacteria_Enterobacteriales_Enterobacteriaceae_Klebsiella_OTU_Otu104	1	0.028552	5	4	19	0	0
Klebsiella-Otu281	Bacteria_Proteobacteria_Gammaproteobacteria_Enterobacteriales_Enterobacteriaceae_Klebsiella_OTU_Otu281	1	0.014631	5	4	5	0	0
Carnimonas-Otu18	Bacteria_Proteobacteria_Gammaproteobacteria_Oceanospirillales_Halomonadaceae_Carnimonas_OTU_Otu18	1	0.091166	3	2	17	0	0
Orbaceae-Otu13	Bacteria_Proteobacteria_Gammaproteobacteria_Orbales_Orbaceae_uncultured_OTU_Otu13	0	0.598334	63	63	1	0.058333	7
Dysgonomonas-Otu9	Bacteria_Bacteroidetes_Bacteroidia_Bacteroidales_Dysgonomonadaceae_Dysgonomonas_OTU_Otu9	0	0.116194	18	18	4	0.088889	4
Gilliamella-Otu17	Bacteria_Proteobacteria_Gammaproteobacteria_Orbales_Orbaceae_Gilliamella_OTU_Otu17	0	0.018955	14	14	1	0	0
Pectobacterium-Otu11	Bacteria_Proteobacteria_Gammaproteobacteria_Enterobacteriales_Enterobacteriaceae_Pectobacterium_OTU_Otu11	0	0.370333	12	12	9	0	0
Chishuiella-Otu12	Bacteria_Bacteroidetes_Bacteroidia_Flavobacteriales_Weeksellaceae_Chishuiella_OTU_Otu12	0	0.289127	10	10	8	0.095238	2
Orbus-Otu2	Bacteria_Proteobacteria_Gammaproteobacteria_Orbales_Orbaceae_Orbus_OTU_Otu2	0	3.153511	7	7	20	0	0
Desulfovibrio-Otu21	Bacteria_Proteobacteria_Deltaproteobacteria_Desulfovibrionales_Desulfovibrionaceae_Desulfovibrio_OTU_Otu21	0	0.022386	6	6	3	0.2	2
Pectobacterium-Otu16	Bacteria_Proteobacteria_Gammaproteobacteria_Enterobacteriales_Enterobacteriaceae_Pectobacterium_OTU_Otu16	0	0.117172	5	5	9	0	0
Candidatus Schmidhempelia-Otu10	Bacteria_Proteobacteria_Gammaproteobacteria_Orbales_Orbaceae_Candidatus Schmidhempelia_OTU_Otu10	0	0.313039	4	4	6	1	1
Dysgonomonas-Otu1	Bacteria_Bacteroidetes_Bacteroidia_Bacteroidales_Dysgonomonadaceae_Dysgonomonas_OTU_Otu1	0	3.773732	4	4	20	0	0
Dysgonomonas-Otu15	Bacteria_Bacteroidetes_Bacteroidia_Bacteroidales_Dysgonomonadaceae_Dysgonomonas_OTU_Otu15	0	0.024452	3	3	5	1	1
Pectobacterium-Otu20	Bacteria_Proteobacteria_Gammaproteobacteria_Enterobacteriales_Enterobacteriaceae_Pectobacterium_OTU_Otu20	0	0.076783	3	3	7	0	0
Orbus-Otu26	Bacteria_Proteobacteria_Gammaproteobacteria_Orbales_Orbaceae_Orbus_OTU_Otu26	0	0.307945	3	3	6	1	1
Enterobacter-Otu379	Bacteria_Proteobacteria_Gammaproteobacteria_Enterobacteriales_Enterobacteriaceae_Enterobacter_OTU_Otu379	0	0.156713	3	3	8	0.333333	1
Enterobacteriaceae-Otu4	Bacteria_Proteobacteria_Gammaproteobacteria_Enterobacteriales_Enterobacteriaceae_Ambiguous_taxa_OTU_Otu4	0	2.409793	2	2	20	0	0

Orbus-Otu53	Bacteria_Proteobacteria_Gammaproteobacteria_Orbales_Orbaceae_Orbus_OTU_Otu53	0	0.034385	2	2	19	0	0
Vagococcus-Otu3	Bacteria_Firmicutes_Bacilli_Lactobacillales_Enterococcaceae_Vagococcus_OTU_Otu3	0	0.534421	2	2	20	0	0
Providencia-Otu5	Bacteria_Proteobacteria_Gammaproteobacteria_Enterobacteriales_Enterobacteriaceae_Providencia_OTU_Otu5	0	0.363557	2	2	20	0	0
Morganella-Otu19	Bacteria_Proteobacteria_Gammaproteobacteria_Enterobacteriales_Enterobacteriaceae_Morganella_OTU_Otu19	0	0.010446	1	1	5	0	0

Network statistics Rajshahi

Label	lineage	posdegree	abundance	degree	negdegree	sample count	clustering	triangles
Klebsiella-Otu281	Bacteria_Proteobacteria_Gammaproteobacteria_Enterobacteriales_Enterobacteriaceae_Klebsiella_OTU_Otu281	1	0.010356	15	14	2	0.066667	1
Enterobacter-Otu123	Bacteria_Proteobacteria_Gammaproteobacteria_Enterobacteriales_Enterobacteriaceae_Enterobacter_OTU_Otu123	1	0.014141	12	11	5	0.3	3
Klebsiella-Otu280	Bacteria_Proteobacteria_Gammaproteobacteria_Enterobacteriales_Enterobacteriaceae_Klebsiella_OTU_Otu280	2	0.016626	11	9	3	0	0
Dysgonomonas-Otu15	Bacteria_Bacteroidetes_Bacteroidia_Bacteroidales_Dysgonomonadaceae_Dysgonomonas_OTU_Otu15	0	0.285576	10	10	9	0	0
Candidatus Schmidhempelia-Otu10	Bacteria_Proteobacteria_Gammaproteobacteria_Orbales_Orbaceae_Candidatus Schmidhempelia_OTU_Otu10	7	0.215617	8	1	5	1	1
Pectobacterium-Otu20	Bacteria_Proteobacteria_Gammaproteobacteria_Enterobacteriales_Enterobacteriaceae_Pectobacterium_OTU_Otu20	0	0.050822	6	6	6	0	0
Pectobacterium-Otu11	Bacteria_Proteobacteria_Gammaproteobacteria_Enterobacteriales_Enterobacteriaceae_Pectobacterium_OTU_Otu11	1	0.203267	6	5	9	0	0
Desulfovibrio-Otu6	Bacteria_Proteobacteria_Deltaproteobacteria_Desulfovibrionales_Desulfovibrionaceae_Desulfovibrio_OTU_Otu6	4	0.399193	6	2	7	0.333333	2
Desulfovibrio-Otu59	Bacteria_Proteobacteria_Deltaproteobacteria_Desulfovibrionales_Desulfovibrionaceae_Desulfovibrio_OTU_Otu59	3	0.093074	5	2	6	0.333333	2
Dysgonomonas-Otu1	Bacteria_Bacteroidetes_Bacteroidia_Bacteroidales_Dysgonomonadaceae_Dysgonomonas_OTU_Otu1	0	4.08075	4	4	20	0	0
Orbus-Otu26	Bacteria_Proteobacteria_Gammaproteobacteria_Orbales_Orbaceae_Orbus_OTU_Otu26	0	0.354554	4	4	7	0	0
Chishuiella-Otu12	Bacteria_Bacteroidetes_Bacteroidia_Flavobacteriales_Weeksellaceae_Chishuiella_OTU_Otu12	0	0.194903	4	4	9	0	0
Klebsiella-Otu47	Bacteria_Proteobacteria_Gammaproteobacteria_Enterobacteriales_Enterobacteriaceae_Klebsiella_OTU_Otu47	3	0.182394	4	1	20	1	1

Klebsiella-Otu72	Bacteria_Proteobacteria_Gammaproteobacteria_Enterobacteriales_Enterobacteriaceae_Klebsiella_OTU_Otu72	4	0.390258	4	0	12	0	0
Desulfovibrio-Otu49	Bacteria_Proteobacteria_Deltaproteobacteria_Desulfovibrionales_Desulfovibrionaceae_Desulfovibrio_OTU_Otu49	2	0.03046	4	2	7	0.333333	1
Orbus-Otu2	Bacteria_Proteobacteria_Gammaproteobacteria_Orbales_Orbaceae_Orbus_OTU_Otu2	3	2.083745	3	0	20	1	1
Orbus-Otu40	Bacteria_Proteobacteria_Gammaproteobacteria_Orbales_Orbaceae_Orbus_OTU_Otu40	3	0.073534	3	0	20	1	1
Orbus-Otu53	Bacteria_Proteobacteria_Gammaproteobacteria_Orbales_Orbaceae_Orbus_OTU_Otu53	2	0.022531	3	1	19	0	0
Gilliamella-Otu17	Bacteria_Proteobacteria_Gammaproteobacteria_Orbales_Orbaceae_Gilliamella_OTU_Otu17	2	0.088822	3	1	6	0	0
Shimwellia-Otu52	Bacteria_Proteobacteria_Gammaproteobacteria_Enterobacteriales_Enterobacteriaceae_Shimwellia_OTU_Otu52	1	0.019658	3	2	13	0.333333	1
Dysgonomonas-Otu9	Bacteria_Bacteroidetes_Bacteroidia_Bacteroidales_Dysgonomonadaceae_Dysgonomonas_OTU_Otu9	0	0.643218	2	2	6	0	0
Klebsiella-Otu104	Bacteria_Proteobacteria_Gammaproteobacteria_Enterobacteriales_Enterobacteriaceae_Klebsiella_OTU_Otu104	1	0.035231	2	1	20	0	0
Escherichia-Shigella-Otu319	Bacteria_Proteobacteria_Gammaproteobacteria_Enterobacteriales_Enterobacteriaceae_Escherichia-Shigella_OTU_Otu319	2	0.015398	2	0	18	1	1
Carnimonas-Otu18	Bacteria_Proteobacteria_Gammaproteobacteria_Oceanospirillales_Halomonadaceae_Carnimonas_OTU_Otu18	0	0.007168	2	2	7	0	0
Citrobacter-Otu7	Bacteria_Proteobacteria_Gammaproteobacteria_Enterobacteriales_Enterobacteriaceae_Citrobacter_OTU_Otu7	1	2.541151	2	1	20	0	0
Candidatus Schmidhempelia-Otu200	Bacteria_Proteobacteria_Gammaproteobacteria_Orbales_Orbaceae_Candidatus Schmidhempelia_OTU_Otu200	1	0.014932	2	1	9	0	0
Dysgonomonas-Otu22	Bacteria_Bacteroidetes_Bacteroidia_Bacteroidales_Dysgonomonadaceae_Dysgonomonas_OTU_Otu22	2	0.036759	2	0	4	0	0
Desulfovibrio-Otu21	Bacteria_Proteobacteria_Deltaproteobacteria_Desulfovibrionales_Desulfovibrionaceae_Desulfovibrio_OTU_Otu21	1	0.022862	2	1	3	0	0
Orbus-Otu8	Bacteria_Proteobacteria_Gammaproteobacteria_Orbales_Orbaceae_Orbus_OTU_Otu8	1	0.308696	2	1	6	0	0
Pectobacterium-Otu16	Bacteria_Proteobacteria_Gammaproteobacteria_Enterobacteriales_Enterobacteriaceae_Pectobacterium_OTU_Otu16	0	0.167403	1	1	6	0	0
Enterobacter-Otu33	Bacteria_Proteobacteria_Gammaproteobacteria_Enterobacteriales_Enterobacteriaceae_Enterobacter_OTU_Otu33	1	0.380868	1	0	15	0	0
Orbus-Otu119	Bacteria_Proteobacteria_Gammaproteobacteria_Orbales_Orbaceae_Orbus_OTU_Otu119	1	0.046867	1	0	16	0	0

Empedobacter-Otu302	Bacteria_Bacteroidetes_Bacteroidia_Flavobacteriales_Weeksellaceae_Empedobacter_OTU_Otu302	1	0.01627	1	0	19	0	0
Empedobacter-Otu97	Bacteria_Bacteroidetes_Bacteroidia_Flavobacteriales_Weeksellaceae_Empedobacter_OTU_Otu97	1	0.023464	1	0	19	0	0
Morganella-Otu19	Bacteria_Proteobacteria_Gammaproteobacteria_Enterobacteriales_Enterobacteriaceae_Morganella_OTU_Otu19	1	0.103113	1	0	5	0	0
Enterobacter-Otu379	Bacteria_Proteobacteria_Gammaproteobacteria_Enterobacteriales_Enterobacteriaceae_Enterobacter_OTU_Otu379	0	0.022227	1	1	2	0	0
